# Characterization and engineering of a two-enzyme system for plastics depolymerization

**DOI:** 10.1073/pnas.2006753117

**Published:** 2020-09-28

**Authors:** Brandon C. Knott, Erika Erickson, Mark D. Allen, Japheth E. Gado, Rosie Graham, Fiona L. Kearns, Isabel Pardo, Ece Topuzlu, Jared J. Anderson, Harry P. Austin, Graham Dominick, Christopher W. Johnson, Nicholas A. Rorrer, Caralyn J. Szostkiewicz, Valérie Copié, Christina M. Payne, H. Lee Woodcock, Bryon S. Donohoe, Gregg T. Beckham, John E. McGeehan

**Affiliations:** ^a^Renewable Resources and Enabling Sciences Center, National Renewable Energy Laboratory, Golden, CO 80401;; ^b^Centre for Enzyme Innovation, School of Biological Sciences, Institute of Biological and Biomedical Sciences, University of Portsmouth, Portsmouth PO1 2DY, United Kingdom;; ^c^Department of Chemical and Materials Engineering, University of Kentucky, Lexington, KY 40506;; ^d^Department of Chemistry, University of South Florida, Tampa, FL 33620;; ^e^Department of Chemistry and Biochemistry, Montana State University, Bozeman, MT 59717;; ^f^Biosciences Center, National Renewable Energy Laboratory, Golden, CO 80401

**Keywords:** polyethylene terephthalate, recycling, upcycling, biodegradation, serine hydrolase

## Abstract

Plastics pollution represents a global environmental crisis. In response, microbes are evolving the capacity to utilize synthetic polymers as carbon and energy sources. Recently, *Ideonella sakaiensis* was reported to secrete a two-enzyme system to deconstruct polyethylene terephthalate (PET) to its constituent monomers. Specifically, the *I. sakaiensis* PETase depolymerizes PET, liberating soluble products, including mono(2-hydroxyethyl) terephthalate (MHET), which is cleaved to terephthalic acid and ethylene glycol by MHETase. Here, we report a 1.6 Å resolution MHETase structure, illustrating that the MHETase core domain is similar to PETase, capped by a lid domain. Simulations of the catalytic itinerary predict that MHETase follows the canonical two-step serine hydrolase mechanism. Bioinformatics analysis suggests that MHETase evolved from ferulic acid esterases, and two homologous enzymes are shown to exhibit MHET turnover. Analysis of the two homologous enzymes and the MHETase S131G mutant demonstrates the importance of this residue for accommodation of MHET in the active site. We also demonstrate that the MHETase lid is crucial for hydrolysis of MHET and, furthermore, that MHETase does not turnover mono(2-hydroxyethyl)-furanoate or mono(2-hydroxyethyl)-isophthalate. A highly synergistic relationship between PETase and MHETase was observed for the conversion of amorphous PET film to monomers across all nonzero MHETase concentrations tested. Finally, we compare the performance of MHETase:PETase chimeric proteins of varying linker lengths, which all exhibit improved PET and MHET turnover relative to the free enzymes. Together, these results offer insights into the two-enzyme PET depolymerization system and will inform future efforts in the biological deconstruction and upcycling of mixed plastics.

## Significance

Deconstruction of recalcitrant polymers, such as cellulose or chitin, is accomplished in nature by synergistic enzyme cocktails that evolved over millions of years. In these systems, soluble dimeric or oligomeric intermediates are typically released via interfacial biocatalysis, and additional enzymes often process the soluble intermediates into monomers for microbial uptake. The recent discovery of a two-enzyme system for polyethylene terephthalate (PET) deconstruction, which employs one enzyme to convert the polymer into soluble intermediates and another enzyme to produce the constituent PET monomers (MHETase), suggests that nature may be evolving similar deconstruction strategies for synthetic plastics. This study on the characterization of the MHETase enzyme and synergy of the two-enzyme PET depolymerization system may inform enzyme cocktail-based strategies for plastics upcycling.

Synthetic polymers pervade all aspects of modern life, due to their low cost, high durability, and impressive range of tunability. Originally developed to avoid the use of animal-based products, plastics have now become so widespread that their leakage into the biosphere and accumulation in landfills is creating a global-scale environmental crisis. Indeed, plastics have been found widespread in the world’s oceans (1–4), in the soil (5), and more recently, microplastics have been observed entrained in the air (6). The leakage of plastics into the environment on a planetary scale has led to the subsequent discovery of multiple biological systems able to convert man-made polymers for use as a carbon and energy source (7–12). These plastic-degrading systems offer a starting point for biotechnology applications toward a circular materials economy (12–16).

Among synthetic polymers manufactured today, polyethylene terephthalate (PET) is the most abundant polyester, which is made from petroleum-derived terephthalic acid (TPA) and ethylene glycol (EG). Given the prevalence of esterase enzymes in nature, PET biodegradation has been studied for nearly two decades, with multiple cutinase enzymes reported to perform depolymerization (17–26). In 2016, Yoshida et al. (10) reported the discovery and characterization of the soil bacterium, *Ideonella sakaiensis* 201-F6, which employs a two-enzyme system to depolymerize PET to TPA and EG, which are further catabolized as a carbon and energy source. Characterization of *I. sakaiensis* revealed the PETase enzyme, which is a cutinase-like serine hydrolase that attacks the PET polymer, liberating bis-(hydroxyethyl) terephthalate (BHET), mono(2-hydroxyethyl) terephthalate (MHET), and TPA. PETase cleaves BHET to MHET and EG, and the soluble MHET product is further hydrolyzed by MHETase to produce TPA and EG. Multiple crystal structures and biochemical studies of *I. sakaiensis* PETase (27–33) revealed an open active-site architecture that is able to bind to PET oligomers. The PETase enzyme likely follows the canonical serine hydrolase catalytic mechanism (34), but open questions remain regarding the mobility of certain residues during the catalytic cycle (27).

Conversely, the structure and function of the MHETase enzyme is far less characterized, with only two published studies focused on MHETase structure and engineering to date (35, 36). These studies report structures at 2.1 to 2.2 Å resolution, wherein the similarity to ferulic acid esterase (FAE) is noted (37, 38). Informed by these structures, engineering efforts aimed to improve turnover of BHET by MHETase, which is a nonnative substrate of the wild-type. Beyond these studies and the original report of MHETase from Yoshida et al. (10), several questions remain regarding the MHETase mechanism and PETase-MHETase synergy. To that end, here we combine structural, computational, biochemical, and bioinformatics approaches to reveal molecular insights into the MHETase structure, mechanism of hydrolysis, the evolution of MHETase activity from FAEs, and engineering of the two-enzyme system for PET depolymerization.

## Results

### Structural Characterization of MHETase Reveals a Core Domain Similar to That of PETase.

**Fig. 1.**
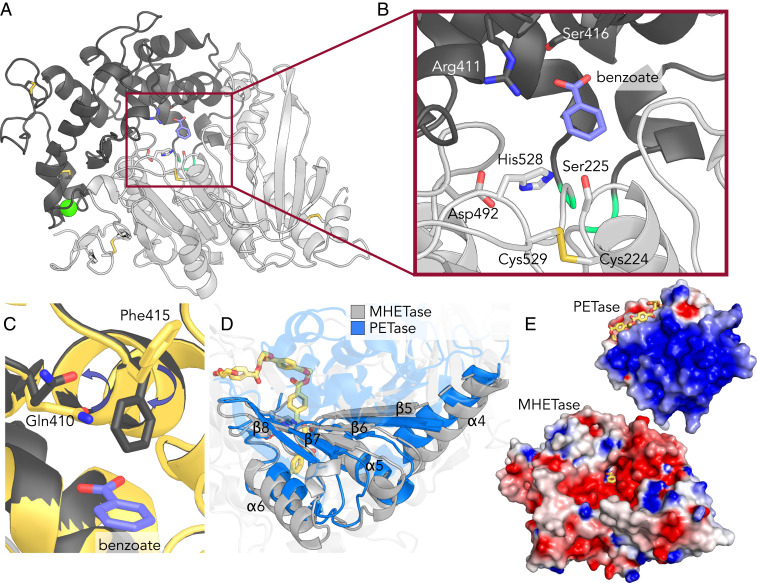
MHETase structural analysis. (*A*) MHETase structure (1.6 Å resolution, PDB ID code 6QZ3) highlighting the catalytic triad, five disulfides (in yellow and gray stick representation), benzoate (purple sticks), and calcium ion (green sphere). The lid domain is dark gray, whereas the hydrolase domain is light gray. Main-chain atoms of the linkage residues Tyr252 and Ala469 are colored lime green (also in *B*). (*B*) Close-up of the MHETase active site with benzoate bound; catalytic triad, active site disulfide, Ser416, and Arg411 shown as sticks. (*C*) The concerted movement of residues Gln410 and Phe415 on ligand binding is illustrated with purple arrows in a superposition of the apo enzyme (yellow) with the ligand bound state (gray). The relative position of benzoic acid is depicted in purple. (*D*) Structural comparison between MHETase (gray) and PETase (PDB ID code 6EQE, in blue), highlighting regions of alignment in the hydrolase domain. A PET tetramer from a prior docking study (29) is shown in yellow sticks (also in *E*). (*E*) Electrostatic potential distribution mapped to the solvent-accessible surface of PETase (29) and MHETase as a colored gradient from red (acidic) at −7 *kT/e* to blue (basic) at 7 *kT/e* (where *k* is the Boltzmann’s constant, *T* is temperature, and *e* is the charge of an electron). PETase is shown with a bound PET tetramer, and MHETase with benzoate bound from the 6QZ3 structure (yellow). The models are drawn to scale and aligned via their catalytic triad demonstrating their relative size difference.

Four crystal structures of MHETase were obtained with the highest-resolution data (6QZ3) extending to 1.6 Å with a benzoate molecule in the active site (Fig. 1 and *SI Appendix*, Table S1). These data reveal a catalytic domain that adopts the α/β-hydrolase fold typical of a serine hydrolase (Fig. 1*A*, light gray), with an extensive lid domain (Fig. 1*A*, dark gray) that partially covers the active site and hosts a well-coordinated Ca^2+^ cation. A similar Ca^2+^ binding site was characterized for *Aspergillus oryzae* FaeB (*Ao*FaeB), wherein it was hypothesized to have a role in stabilizing the lid domain (38). Overall, the structure of MHETase is most similar to that of FAEs, as discussed previously (35, 36). The structural conservation between the hydrolase domains of MHETase and PETase is striking (Fig. 1*D* and *SI Appendix*, Fig. S1), and despite the large insertion of ∼240 residues representing the lid domain, residues Ser225, Asp492, and His528 effectively reconstitute the catalytic triad (Fig. 1*B*). In fact, the terminal residues of the lid domain converge to within hydrogen-bonding distance of each other (Tyr252-Ala469, 2.9 Å), creating a compact linkage to the hydrolase domain. The lid domain of MHETase is exceptionally large, as average lid domains in α/β-hydrolases tend to be ∼100 residues (34), and is more typical of a lid from tannase family members (*vide infra*). The equivalent connection site in PETase is occupied by a seven-residue loop.

In addition, we determined two apo structures with alternative packing (6QZ2 and 6QZ4), one structure with a fully occupied benzoic acid ligand (6QZ3), and one with partially occupied benzoic acid (6QZ1). We observed that residue Phe415 adopts a “closed” orientation on substrate binding consistent with prior substrate bound structures (PDB ID codes 6QGA and 6QGB) (35), and the partially occupied site results in an intermediate dual “open/closed” conformation (*SI Appendix*, Fig. S2 *A*–*C*) (35). The only other amino acid with side-chain positioning correlated with ligand binding is Gln410. When Phe415 is in the open position, the side chain of Gln410 pivots toward the active site to a position wherein the heavy atoms would be as close as 1.8 Å to those of Phe415 if it were in the closed conformation (*SI Appendix*, Fig. S2*D*).

Given the difference in overall isoelectric point (pI) between PETase (9.65) and MHETase (5.11), we generated electrostatic surface profiles for comparison (Fig. 1*E*). As previously reported, PETase has a highly polarized surface charge (29), whereas MHETase exhibits a more heterogeneous and acidic surface charge distribution. MHETase contains five disulfide bonds (Fig. 1*A*). One of the MHETase disulfides is located at the active site, connecting cysteines (Cys224 and Cys529) adjacent to the catalytic residues (Ser225 and His528, respectively), which is conserved in tannase family members (38). PETase lacks a structurally equivalent disulfide, and the aligning residues in PETase (Trp159 and Ser238) are the same two residues mutated by Austin et al. (29) to yield a PETase substrate binding groove similar to that of cutinases, resulting in improved activity on a crystalline PET substrate.

### Molecular Simulations of the MHETase Reaction Predict Deacylation Is Rate-Limiting.

The MHETase structure suggests a serine hydrolase mechanism for MHET hydrolysis (34). To elucidate the detailed reaction mechanism, we first constructed a Michaelis complex in silico utilizing the CHARMM (39) molecular simulation package (details in *SI Appendix*, *Supplementary Materials and Methods*). To examine MHETase dynamics and ligand stability, classic molecular dynamics (MD) simulations were conducted with NAMD (40) (all simulations totaling 2.25 µs) utilizing the CHARMM forcefield (41). Given the observed dual occupancy for Phe415 positioning in the crystal structures, we simulated in triplicate (each simulation 150 ns in length) the four combinations of Phe415 position (open and closed) and active site occupancy (empty active site and MHET-bound). In each case wherein Phe415 begins in the closed state (starting configurations from 6QZ3 structure, with coordinates for residues 56 to 61 from 6QZ4), Phe415 opens in the first 10 ns and rarely returns to the closed state; simulations that begin with Phe415 open (built from 6QZ4 structure) all remain open. To examine the effect of calcium binding, a fifth scenario absent of either MHET or Ca^2+^ was modeled in triplicate 150-ns simulations (the prior four scenarios each include bound Ca^2+^). These trajectories show evidence for lid stabilization upon Ca^2+^ binding mainly in the immediate vicinity of the calcium binding site (*SI Appendix*, Fig. S3). When bound at the active site, the carboxylate motif of MHET exhibits stable hydrogen bonds with Arg411 and Ser416, while the carbonyl is stabilized via hydrogen bonds to the oxyanion hole residues, Glu226 and Gly132 (*SI Appendix*, Figs. S4 and S5). In all three simulations with MHET bound and Phe415 open, MHET maintains these interactions and remains bound at the active site throughout the duration of the 150-ns simulation and primed for hydrolysis (hydrogen bond distance between Ser225 and His528 = 2.0 ± 0.2 Å; nucleophilic attack distance between Ser225 and MHET = 3.1 ± 0.3 Å; hydrogen bond distance between Asp492 and His528 = 1.8 ± 0.1 Å). Further analysis of the MD simulations, including time traces for these important interactions, is available in the *SI Appendix*, *Supplementary Materials and Methods*.

Serine hydrolases catalyze a two-step reaction involving formation of an acyl-enzyme intermediate (acylation) that is released hydrolytically in the second step (deacylation) (34). We utilized the Amber software package (42) to perform hybrid quantum mechanics/molecular mechanics (QM/MM) 2D umbrella sampling with semiempirical force field SCC-DFTB (43) to study the catalytic steps. Judicious selection of a reaction coordinate is critical for kinetically meaningful barrier calculations. We chose the forming and breaking C-O bonds to map the free-energy landscape for both reaction steps, informed by transition path sampling studies of other hydrolase enzymes (44, 45).

**Fig. 2.**
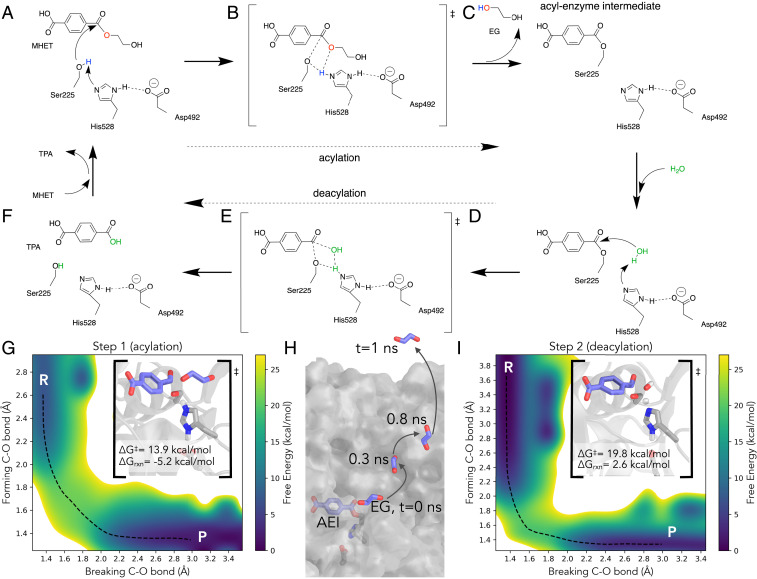
The MHETase catalytic mechanism: (*A*) reactant, (*B*) transition state, and (*C*) product of acylation in which His528 transfers a proton from Ser225 to the EG leaving group. In deacylation (*D*–*F*), His528 plays a similar role and restores the catalytic serine, transferring a proton from a water molecule to Ser225 and generating a free TPA molecule. (*G*) The free-energy surface for acylation computed along a reaction coordinate described by the breaking and forming C-O bonds. The minimum free energy path is shown in black dashes. (*H*) Following acylation, EG leaves the active site within 1 ns of a classic MD simulation. (*I*) The free-energy surface for deacylation, exhibiting a predicted higher barrier than acylation. The MFEP is shown in black dashes.

In acylation, the catalytic serine (Ser225) is deprotonated by His528, activating it for nucleophilic attack upon the carbonyl C of MHET, liberating EG and forming the acyl-enzyme intermediate (AEI) (Fig. 2 *A*–*C* and Movie S1). The minimum free-energy path (MFEP) computed from QM/MM 2D umbrella sampling (along the forming C-O bond between the MHET carbonyl C and Ser225 and the breaking MHET C-O ester bond) predicts an acylation free-energy barrier (∆G^‡^) of 13.9 ± 0.17 kcal/mol with an overall reaction free energy (∆G_reaction_) of −5.2 ± 0.04 kcal/mol (Fig. 2*G* and *SI Appendix*, Fig. S6*A*). Although serine hydrolases have at times been considered to proceed through metastable tetrahedral intermediates along the reaction pathway for acylation and deacylation (46–48), the acylation MFEP calculated from 2D umbrella sampling does not indicate intermediate configurations with metastability.

Following cleavage of EG from MHET, classic MD simulation reveals that EG leaves the active site in the presence of the AEI (Fig. 2*H* and Movie S2). In one simulation, EG initially maintains a hydrogen bond with His528 for ∼100 ps, then dislodges from the active site, and is free in solution within 1 ns. Three identical simulations were initiated, and EG exits the active site within 4 ns in each. An important implication of this observation is that the deacylation reaction proceeds without EG in the active site. This allows greater access for water molecules to approach the charged nitrogen of His528 for deacylation (*SI Appendix*, Fig. S7).

Deacylation involves nucleophilic attack by a water molecule on the AEI, liberating TPA (Fig. 2 *D*–*F* and Movie S3). His528 plays a similar role as in acylation, deprotonating the catalytic water and transferring this proton to the catalytic serine, regenerating Ser225 for another catalytic cycle. The MFEP computed from QM/MM 2D umbrella sampling (along the forming C-O bond between MHET and water and the breaking AEI C-O bond) reveals a deacylation free-energy barrier (∆G^‡^) of 19.8 ± 0.10 kcal/mol and an overall reaction free energy (∆G_reaction_) of 2.6 ± 0.07 kcal/mol (Fig. 2*I* and *SI Appendix*, Fig. S6*B*). Together, the two catalytic steps are exergonic by −2.6 ± 0.08 kcal/mol. Deacylation is predicted to be the rate-limiting step, with a rate of 7.1 ± 1.1 × 10^−2^ s^−1^ (from transition state theory, at 30 °C, and assuming a transmission coefficient of 1), more than four orders-of-magnitude slower than acylation (1.02 ± 0.28 × 10^3^ s^−1^). As in acylation, metastable configurations along the MFEP are not observed.

### Bioinformatics Analysis Suggests That MHETase Evolved from an FAE.

**Fig. 3.**
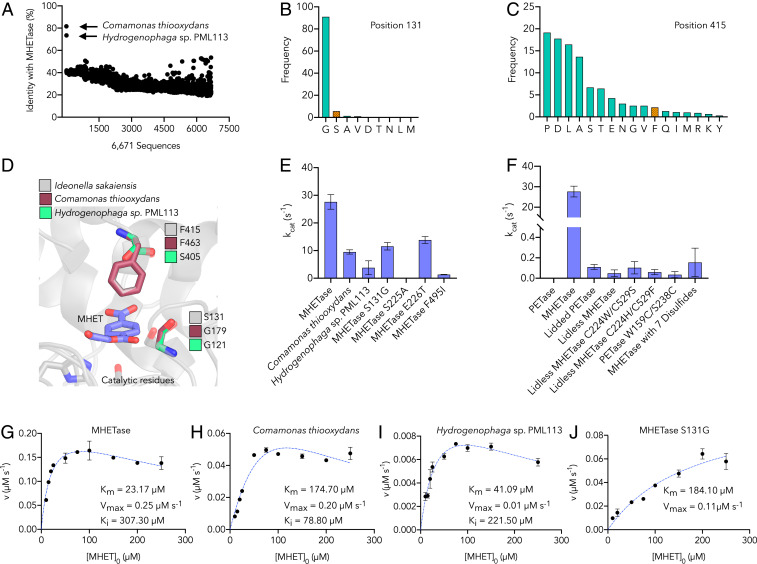
Characterization of MHETase, homologs, and mutants. (*A*) Sequence identity of 6,671 tannase family sequences retrieved by PSI-BLAST compared to MHETase. Sequences (*x* axis) are in the same order returned by PSI-BLAST. (*B* and *C*) Conservation analysis of residue positions 131 (*B*) and 415 (*C*) (using MHETase numbering). Frequency of each amino acid is based on a multiple sequence alignment of the 6,671 tannase family sequences. The residue found in MHETase at each position is indicated in orange. (*D*) Homology model of the MHET-bound active site within 6 Å of the bound substrate comparing MHETase to homology models of the *C. thiooxydans* and *Hydrogenophaga* sp. PML113 homologs (generated by SWISS-MODEL) (54), showing sequence variation at residue positions corresponding to Ser131 and Phe415 in MHETase. (*E* and *F*) The rate of enzymatic turnover of MHET determined for MHETase, both homologous enzymes, and the indicated MHETase mutants, all of which are active on MHET save the catalytic mutant (S225A) (*E*), and enzymatic turnover rates for PETase, MHETase, and selected mutants on MHET (*F*), using 5 nM purified enzyme and 250 µM substrate at 30 °C. (*G*–*J*) The initial enzyme reaction velocity as a function of substrate concentration for MHETase, *C. thiooxydans*, *Hydrogenophaga* sp. PML113, and the MHETase S131G mutant, respectively. Dashed blue lines represent the Michaelis–Menten kinetic model fit with substrate inhibition (*G*–*I*) or fit with the simple Michaelis–Menten model (*J*). Key kinetic parameters are provided in the *Inset*. Additional parameters and confidence intervals on the listed parameters are provided in *SI Appendix*, Table S3.

Beyond structural and mechanistic investigations, we were also interested in understanding potential MHETase evolutionary ancestry and identifying other MHET-active enzymes from natural diversity. MHETase belongs to the tannase family (PFAM ID: PF07519), which consists of fungal and bacterial FAEs, fungal and bacterial tannases, and several bacterial homologs of unknown function (49). To elucidate sequence relationships between MHETase and tannase family enzymes, we performed bioinformatic analyses of 6,671 tannase family sequences retrieved from the National Center for Biotechnology Information (NCBI) via PSI-BLAST (50). MHETase shares low sequence similarity (<53%) with most sequences in the family, with the exception of homologs from *Comamonas thiooxydans* strains DS1, DF1, and DF2 (strain: NCBI:txid363952, protein:GenBank WP_080747404.1) (51) and *Hydrogenophaga* sp. PML113 (strain: NCBI:txid1899350, protein:GenBank WP_083293388.1), which exhibit 81% and 73% identity to MHETase, respectively (Fig. 3*A*). Since initial identification of the homologous *C. thiooxydans* sequence (WP_080747404.1), this entry was removed from GenBank, as discussed in *SI Appendix*, *Supplementary Materials and Methods*.

Using the multiple sequence alignment of 6,671 tannase family sequences, we performed conservation analysis with MHETase sequence positions as a reference (*SI Appendix*, Figs. S8 and S9), which shows that most positions in the active site are highly conserved. Notable exceptions are positions 257, 411, 415, and 416, which exhibit low conservation scores and are less conserved than 80% of MHETase positions overall (*SI Appendix*, Fig. S8 *B* and *C*). It is noteworthy (*vide infra*) that position 131 is a well-conserved glycine in 91% of tannase family sequences but serine appears at position 131 in MHETase (Fig. 3*B*). Furthermore, the 10 cysteine positions in MHETase that form 5 disulfide bonds are highly conserved in the tannase family (*SI Appendix*, Fig. S10*A*). Although a sixth disulfide bond exists in *Ao*FaeB (38), less than 8% of tannase family sequences exhibit this sixth disulfide bond, and the sixth disulfide bond positions are variable among this set (*SI Appendix*, Fig. S10*B*). One cysteine of the sixth disulfide bond in *Ao*FaeB is a single residue variation found in MHETase (38), whereas the other sits on a loop where a 15-residue deletion is found in MHETase.

With this large dataset, we further conducted phylogenetic analysis of 120 sequences selected from tannase family sequences that were clearly annotated as tannases or FAEs in GenBank, including MHETase (*SI Appendix*, Table S2). In the phylogenetic tree (*SI Appendix*, Fig. S11), bacterial and fungal enzymes form paraphyletic groups, and within these groups, there are separate FAE and tannase subgroups. MHETase and the *C. thiooxydans* and *Hydrogenophaga* sp. PML113 homologs are found within a group of proteobacterial FAEs (bootstrap value > 95%). In addition, when separate profile hidden Markov models (pHMM) are constructed with the annotated tannase family FAE and tannase sequences (52), and then aligned with MHETase, a higher alignment score is achieved with the FAE pHMM than with the tannase pHMM (456.8 vs. 396.8), suggesting that MHETase is more similar to FAEs than tannases.

### Biochemical Characterization of Active-Site MHETase Mutants and Homologs Reveals Important Residues for MHET Hydrolytic Activity.

From the bioinformatics analyses, we selected the MHETase homologs from *C. thiooxydans* and *Hydrogenophaga* sp. PML113 (Fig. 3*A*) to test for MHET hydrolysis activity, which, along with the *I. sakaiensis* MHETase, were produced in *Escherichia coli* and purified. Activity assays were performed for each enzyme to determine MHET turnover rates (Fig. 3*E*). The turnover rate (*k*_*cat*_) for MHETase is 27.6 ± 2.6 s^−1^, as compared to 9.5 ± 0.8 s^−1^ and 3.8 ± 2.5 s^−1^ for the *C. thiooxydans* and *Hydrogenophaga* sp. PML113 enzymes, respectively. The enzymes were also evaluated over a range of substrate concentrations to determine the Michaelis–Menten kinetic parameters (Fig. 3 *G*–*J* and *SI Appendix*, Table S3). FAEs have been shown to exhibit concentration-dependent substrate inhibition (53) in addition to the likely product inhibition of the enzyme (35). MHETase and both homologs also display this behavior. Using a substrate inhibition model (details in *SI Appendix*, *Supplementary Materials and Methods*), evaluation of the substrate-dependent reaction kinetics shows that MHETase more efficiently accepts MHET as a substrate than the *C. thiooxydans* and *Hydrogenophaga* sp. PML113 homologs, demonstrated by a *K*_m_ value of 23.17 ± 1.65 µM as compared to values of 174.70 ± 4.75 µM and 41.09 ± 3.38 µM, respectively (Fig. 3 *G*–*I* and *SI Appendix*, Table S3). However, MHETase is also the most susceptible to substrate inhibition with a *K*_k_ value of 307.30 ± 20.65 µM. Despite the difference in affinity for MHET, MHETase and the *C. thiooxydans* enzyme exhibit similar maximal reaction rates, while the enzyme from *Hydrogenophaga* sp. PML113 is slower. The MHETase reaction efficiency, reported as *k*_*cat*_/*K*_m_, is ∼10-fold higher than for the *C. thiooxydans* enzyme and ∼20-fold higher than the *Hydrogenophaga* sp. PML113 enzyme.

Homology models of both the *C. thiooxydans* and *Hydrogenophaga* sp. PML113 enzymes were constructed with SWISS-MODEL (54) using the MHETase structure as a template (PDB ID code 6QZ3), and the active site aligned with a modeled MHET-bound MHETase structure (Fig. 3*D*). As noted, position 131 is a serine in MHETase, but a glycine in the two homologs (*C. thiooxydans*, Gly179 and *Hydrogenophga* sp. PML113, Gly121) (Fig. 3*B*). The *C. thiooxydans* enzyme is otherwise identical within 6 Å of the docked MHET ligand, whereas the *Hydrogenophaga* sp. PML113 enzyme also exhibits a serine in the equivalent position to the MHETase residue Phe415 (Fig. 3*C*). An S131G mutant of MHETase was constructed to examine the role of this residue in MHET hydrolytic activity, and steady-state enzyme kinetics were evaluated. The MHETase S131G mutant does not demonstrate concentration-dependent substrate inhibition, as is observed for the wild-type enzyme, which is likely due to the poor affinity for MHET. The S131G mutant has a *K*_m_ value approximately eightfold higher than that of wild-type MHETase and the reaction efficiency is reduced to ∼3% that of the wild-type (Fig. 3*J* and *SI Appendix*, Table S3), illustrating the importance of this residue in MHET turnover.

Focusing on residues within the coordination sphere of the docked MHET ligand, amino acids and their frequencies across the tannase family were compared to MHETase (*SI Appendix*, Figs. S8 and S9). In position 495, MHETase features a phenylalanine, while isoleucine is also a common residue in this position across the tannase family. Palm et al. (35) demonstrated that Phe495 has a modest effect on activity by mutation to alanine. We constructed and evaluated an MHETase mutant with isoleucine in this position (F495I), which dramatically impairs activity, lowering the turnover rate from 27.6 ± 2.6 s^−1^ to 1.3 ± 0.7 s^−1^ (Fig. 3*E*). In position 226, which is part of the conserved “lipase box” motif in serine hydrolases (55), MHETase exhibits a glutamate, while threonine and asparagine are more common among tannase family members. Mutation of this lipase box residue to threonine (E226T) yielded a ∼50% reduction in MHET activity relative to the wild-type MHETase (Fig. 3*E*). Mutation of the catalytic serine (S225A), as expected, produced an inactive enzyme.

### Unique Structural Features between MHETase and PETase Determine Substrate Specificity and Stability.

Given the structural similarities of the MHETase and PETase core domains (Fig. 1 *C* and *D*), we were interested in understanding the role of unique MHETase features, namely the lid domain and the active site disulfide bond between Cys224 and Cys529, on substrate specificity and MHET hydrolytic activity. Accordingly, the lid was both added to PETase (“lidded PETase”) and removed from MHETase (“lidless MHETase”). Given the natural substrate specificities of wild-type PETase and MHETase, we hypothesized that the former could confer MHET activity, but abolish PET hydrolytic potential, whereas the latter was expected to have the opposite effect. The lidded PETase was created by replacing the seven-residue loop of PETase (Trp185:Phe191, PETase numbering) with Gly251:Thr472 from MHETase. In control experiments, wild-type PETase exhibited no detectable activity on MHET, and the lidded PETase is not able to degrade amorphous PET film. However, meager activity of lidded PETase was detected on MHET (*k*_*cat*_ = 0.11 ± 0.02 s^−1^) (Fig. 3*F*). The lidless MHETase was created by replacing the MHETase lid (Gly251:Thr472) with the seven-residue loop of PETase (Trp185:Phe191, PETase numbering). This construction results in an exposed MHETase active site, possibly allowing for acquired PET hydrolytic activity. The resulting enzyme has a *k*_*cat*_ value on MHET of 0.05 ± 0.03 s^−1^, 1,000-fold lower than the rate for wild-type MHETase, demonstrating that the lid domain is crucial for MHET hydrolytic activity (Fig. 3*F*). The lidless MHETase enzyme was also unable to degrade amorphous PET film over 96 h, despite the more accessible active site.

Similarly, variants of lidless MHETase were generated to remove the active site disulfide and replace the two sites with tryptophan and serine (lidless MHETase C224W/C529S) (*SI Appendix*, Fig. S1*B*) to reconstitute the PETase active site, or with histidine and phenylalanine (lidless MHETase C224H/C529F), matching the active site of the double-mutant PETase variant previously shown to exhibit improved PET hydrolytic activity on crystalline PET (29). The lidless MHETase C224W/C529S mutant, which reconstitutes the wild-type active site motif of PETase, displays the same turnover rate (within error) as the lidded PETase mutant on MHET (*k*_*cat*_ *=* 0.10 ± 0.06 s^−1^), while the lidless MHETase C224H/C529F mutant is even less active on MHET (*k*_*cat*_ *=* 0.06 ± 0.03 s^−1^) (Fig. 3*F*). We also generated a PETase variant to recreate the active site disulfide found in MHETase (PETase W159C/S238C). The PETase mutant exhibited very low MHET hydrolytic activity (*k*_*cat*_ **=** 0.03 ± 0.3 s^−1^), and similarly had no activity on BHET or amorphous PET film.

To delineate the effects of engineering the lid and removing the active site disulfide bond, we also generated three MHETase mutants altering only the active site disulfide motif (C224A/C529A, C224W/C529S, and C224H/C529F). We hypothesized that removal of this disulfide bond may diminish the thermal stability of MHETase. However, each of these variants either expressed in inclusion bodies or did not express at all. Attempts were also made to introduce disulfide motifs into MHETase that are found in PETase (G489C/S530C) or in *Ao*FaeB. To recapitulate the *Ao*FaeB disulfide, the mutations include both a point mutation (S136C) as well as the insertion of a 15-residue loop from *Ao*FaeB that harbors the partnering cysteine residue. As with the active site disulfide mutants, these mutants either expressed in inclusion bodies or did not express at all. A variant was also created that included both the PETase-like disulfide (G489C/S530C) and the *Ao*FaeB modification (S136C with 15-residue loop from *Ao*FaeB). This last variant, with seven total disulfides, was successfully expressed and had very low activity on MHET (*k*_*cat*_ *=* 0.16 ± 0.14 s^−1^) (Fig. 3*F*). A full list of clones, mutants, and primers can be found in Dataset S1.

### MHETase Is Catalytically Inactive on MHE-Isophthalate and MHE-Furanoate.

We evaluated the substrate specificity of MHETase using the monohydroxyethyl monomer unit of two additional compounds. Specifically, assays were performed with mono(2-hydroxyethyl)-isophthalate (MHEI) (*SI Appendix*, Fig. S12) and mono(2-hydroxyethyl)-furanoate (MHEF) (*SI Appendix*, Fig. S13). Isophthalate is a common comonomer in industrial PET formulations used to modify crystallinity, such that MHEI could be released from polyester depolymerization. PETase has been demonstrated to deconstruct other aromatic polyesters (29), including polyethylene furanoate (PEF), yielding MHEF as a product of the enzymatic hydrolysis reaction. Over the course of 24 h at 30 °C, no MHETase activity was detected for either substrate using substrate concentrations from 25 to 250 μM, in contrast with complete hydrolysis of MHET (*SI Appendix*, Fig. S14) in the same time using identical reaction conditions.

To explain the inability of MHETase to act on MHEI and MHEF, we conducted flexible ligand/flexible receptor docking simulations and predicted 10 binding orientations for each molecule (MHET, MHEI, and MHEF) in MHETase. These docking simulations indicate that MHET binds to MHETase with a binding free energy of −7.13 kcal/mol and in a catalytically primed configuration. This binding mode features the carbonyl C of MHET within 3.2 Å of Ser225-O, which itself is within 2.90 Å of His528-N(e) and His528-N(d) is 3.93 Å from Asp492-O (*SI Appendix*, Fig. S15). For MHEI and MHEF, no binding modes were predicted that exhibit similarly favorable binding free energies, feature the MHET carbonyl C within range for attack by Ser225, and stabilize the carbonyl of the ester in the oxyanion hole, suggesting that MHETase will not readily act on these molecules.

### PETase and MHETase Act Synergistically during PET Depolymerization.

**Fig. 4.**
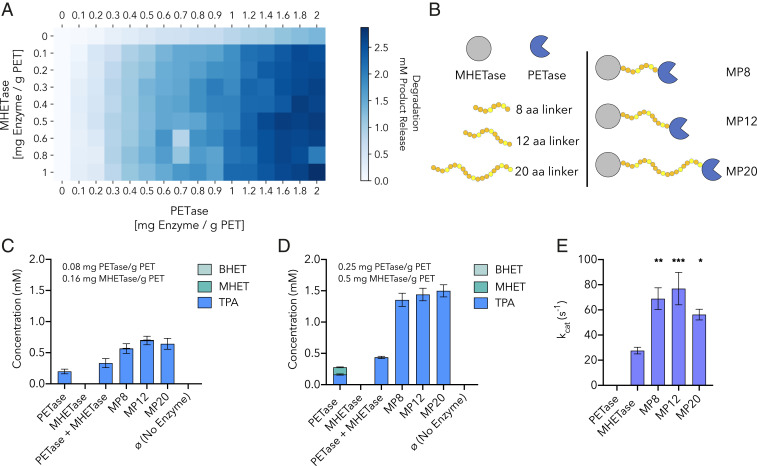
PETase-MHETase synergy and chimeric enzymes. (*A*) Heatmap of synergistic degradation by PETase and MHETase on amorphous PET film over 96 h at 30 °C. Total product release in millimolars (sum of BHET, MHET, and TPA); *x* axis: PETase loading (mg/g PET), *y* axis: MHETase loading (mg/g PET). (*B*) Illustrations of three chimeric enzymes. Linkers composed of glycine (orange) and serine (yellow) residues connecting the C terminus of MHETase to the N terminus of PETase. (*C* and *D*) Comparison of depolymerization performance of PETase alone, MHETase alone, PETase and MHETase at equimolar loading, and the three chimeric enzymes on amorphous PET film after 96 h at 30 °C. Product release in millimolars resulting from hydrolysis by (*C*) 0.08 mg PETase/g PET or 0.16 mg MHETase/g PET and (*D*) 0.25 mg PETase/g PET or 0.5 mg MHETase/g PET. All comparisons are statistically significant with *P* ≤ 0.0001 based on two-way ANOVA analysis and Tukey’s multiple comparisons test. (*E*) MHET turnover rate by each chimeric enzyme compared to MHETase alone using 250 µM MHET and 5 nM enzyme. Asterisks indicate statistically significant comparisons between MHETase and each chimera enzyme with *P ≤ 0.01, **P ≤ 0.001, and ***P ≤ 0.0005.

While MHET is susceptible to hydrolysis by a number of PET-degrading cutinases, *I. sakaiensis* requires the action of two enzymes for PET degradation to liberate TPA and EG (10). Given the turnover rates for MHETase reported here, depolymerization by PETase is likely the rate-limiting step when the enzymes are employed together. To investigate the action of the two-enzyme system, we thus measured the extent of hydrolysis of a commercial amorphous PET substrate over 96 h at 30 °C using PETase and MHETase at varying concentrations (Fig. 4*A* and *SI Appendix*, Table S4). As expected, MHETase alone has no activity on PET film. Over the range of enzyme loadings tested (0 to 2.0 mg enzyme/g PET), degradation by PETase alone, as determined by concentration of product released (the sum of BHET, MHET, and TPA), scales with enzyme loading. Upon addition of MHETase in the reaction, at any loading tested (0.1 to 1.0 mg MHETase/g PET), product release still scales with PETase loading, but at a markedly higher level than with PETase alone (Fig. 4*A*). The overall trend of degradation within the range of enzyme loadings tested, which shows increasing levels of constituent monomers released as concentration of both enzymes increases, is indicative that these reactions are enzyme-limited under these conditions, rather than substrate-limited. The synergy study does not strongly indicate that any particular ratio of PETase to MHETase results in optimal degradation over the enzyme loadings tested, but rather that degradation scales with PETase loading and the presence of MHETase, even at low concentrations relative to PETase, improves total degradation.

### Chimeric Proteins of MHETase and PETase Improves PET Degradation and MHET Hydrolysis Rates.

In light of the highly synergistic relationship between PETase and MHETase on amorphous PET, where increasing loading of each enzyme results in more constituent monomer release, we next examined how proximity of the two enzymes influences hydrolytic activity. Chimeric proteins covalently linking the C terminus of MHETase to the N terminus of PETase, using flexible glycine-serine linkers of 8, 12, and 20 total glycine and serine residues, were generated and assayed for degradation of amorphous PET (Fig. 4*B*). Varying linker lengths were explored to understand the effect of increased mobility between the two domains (56). Furthermore, two enzyme loadings were compared: The lower loading corresponding to ∼0.08 mg PETase/g PET and 0.16 mg MHETase/g PET and the higher enzyme loading corresponding to 0.25 mg PETase/g PET and 0.5 mg MHETase/g PET (Fig. 4 *C* and *D*). At both loadings, when comparing the extent of degradation achieved by PETase alone, MHETase alone, and an equimolar mix of PETase and MHETase, the chimeric proteins outperform PETase, as well as the mixed reaction containing both PETase and MHETase unlinked in solution. Furthermore, the chimeras demonstrate a higher catalytic activity on MHET (Fig. 4*E*). Chimeric constructs linking the C terminus of PETase to the N terminus of MHETase did not successfully express protein (Fig. 4*B*). SEM analysis of digested amorphous PET film confirms degradation (*SI Appendix*, Fig. S16).

## Discussion

The ability to degrade polymers to their monomeric units is important for subsequent reuse in new products, which is a critical technical advance needed to enable a global circular materials economy. In biological systems, complete depolymerization to monomers can be necessary for microbial uptake and growth, as in *I. sakaiensis* wherein MHETase is the enzymatic partner to PETase, together allowing for the complete degradation of PET to TPA and EG for catabolism (10). Prior studies presenting MHETase crystal structures focused upon understanding and tuning substrate specificity, particularly the rational engineering of MHETase to impart BHET hydrolysis activity (35, 36). Drawing inspiration from our structural analyses, this complementary study offers further insights into the two-enzyme PETase/MHETase system.

The recent structural report from Palm et al. (35) highlighted several important amino acid contributions to substrate specificity in MHETase, specifically focusing on active site residues. Of note, they pointed out the importance of Phe415 for substrate binding via an “induced fit” mechanism and highlighted Arg411 with respect to hydrogen bonding of the MHET carboxylate group, both of which are proposed to be drivers of substrate specificity. In addition, beyond engineering a starting point for BHET activity in MHETase for further optimization, the potential for MHEF turnover was suggested, given the proposed utility of PEF as a bio-based PET replacement (57). In our previous work (29), we demonstrated that PETase effectively depolymerizes PEF, but the results here do not indicate the same for MHETase on MHEF, and docking simulations agree with the observed patterns in MHETase selectivity. Despite success with predicting a low-energy catalytically competent binding mode for MHET to MHETase, we were only able to predict one binding mode of MHEF to MHETase with the MHEF nucleophilic carbonyl in the oxyanion hole, but in this pose the MHEF carboxylate moiety is not in range to interact with Arg411, suggesting that further active site engineering will be necessary to enable MHEF turnover. Similarly, only one binding mode for MHEI was predicted wherein the catalytic triad was oriented for catalysis but, akin to MHEF, the nonlinearity of the molecule prevents simultaneous interaction with the oxyanion hole and R411.

The enzyme kinetics studies presented here reveal a substantial reduction in activity for the S131G, E226T, and F495I MHETase mutants, indicating that these positions play important roles in substrate specificity and catalytic efficiency. A previous study also demonstrated greatly reduced hydrolytic activity by a F415S variant (36). Additionally, two homologs identified via bioinformatics analysis from *C. thiooxydans* and *Hydrogenophaga* sp. PML113 exhibit extremely similar active site environments (Fig. 3*D*), with the only exceptions being variations at positions 131 and 415 (MHETase numbering), and these homologs display reaction efficiencies (*k*_*cat*_/*K*_m_) reduced by an order-of-magnitude (*SI Appendix*, Table S3). Furthermore, as the amino acids at these positions in wild-type MHETase are less common in tannase family sequences (*SI Appendix*, Fig. S9), and mutation to the more common amino acids led to a reduction in activity, this suggests that these two sequence positions were specifically evolved in MHETase to accommodate MHET.

Two-enzyme systems for complete PET degradation have been examined previously, either derived from a single microorganism (e.g., *Thermobifida fusca*) (58) or screened from multiple sources for optimal activity (25, 59). The enzyme synergy results for the *I. sakaiensis* PETase/MHETase system on amorphous PET display a clear performance improvement when MHETase is included in the reaction. Namely, overall degradation scales with PETase loading within the tested range (0 to 2.0 mg PETase/g PET), but the inclusion of MHETase in the degradation reaction markedly improves depolymerization and this synergistic enhancement also scales with MHETase loading.

The presence of confirmed MHETase homologs in *C. thiooxydans* and *Hydrogenophaga* sp. PML113 suggests that these bacteria may harbor abilities for TPA catabolism (*SI Appendix*, Fig. S17). Bioinformatics analysis was thus conducted to query the genomes of the strains compared to known TPA catabolic genes from *I. sakaiensis* (10), *Comamonas* sp. E6 (60, 61), *Delftia tsuruhatensis* (62), *Paraburkholderia xenovorans* (63), *Rhodococcus jostii* RHA1 (64), and *Rhodococcus* sp. DK17 (65), including putative PETases, terephthalate transporter genes, two-component terephthalate dioxygenases, the 1,2-dihydroxy-3,5-cyclohexadiene-1,4-dicarboxylate dehydrogenase, and the three types of protocatechuate (PCA) dioxygenases (PCA-2,3, PCA-3,4, and PCA-4,5-dioxygenases) (*SI Appendix*, Table S5). This analysis revealed that neither *C. thiooxydans* nor *Hydrogenophaga* sp. PML113 harbor putative PETase genes. Interestingly, both strains exhibit genes encoding for TPA catabolic enzymes and transporters highly homologous to those of *I. sakaiensis*, *Comamonas* sp. E6, and *D. tsuruhatensis* (in all cases above 60% identity) (*SI Appendix*, Figs. S18 and S19), suggesting that they are highly likely able to turnover TPA to PCA, a common central intermediate in aerobic aromatic catabolic pathways (66). Each strain also contains annotated PCA-4,5-dioxygenases (*SI Appendix*, Table S5). Further experimental work will be required to understand if either of these bacteria exhibit the ability to depolymerize PET, perhaps through another type of mechanism than via ester hydrolases, or perhaps like *Comamonas* sp. E6, they are primarily able to consume soluble, xenobiotic intermediates. Perhaps these strains could serve as useful sources of TPA catabolic genes for synthetic biology efforts associated with biological plastics recycling and upcycling (67).

The enzymatic deconstruction of recalcitrant natural polymers, such as cellulose, hemicellulose, and chitin, is accomplished in nature by the action of mixtures of synergistic enzymes secreted from microbes (68, 69). For example, as observed in fungal cellulase systems for cellulose depolymerization, these mixtures typically contain a subset of enzymes to act directly on solid polymeric substrates via interfacial enzyme mechanisms, and complementary enzymes (e.g., β-glucosidases) that further process solubilized intermediates to monomeric constituents (e.g., cellobiose hydrolysis to glucose). Given that natural microbial systems evolved over millions of years to optimally degrade recalcitrant polymers, perhaps it is thus not surprising, in hindsight, that a soil bacterium such as *I. sakaiensis* evolved the ability to utilize a crystalline polyester substrate with, to our collective knowledge, a two-enzyme system (10, 16). Extending the analogy of cellulase enzymes and plant cell wall deconstruction for breaking down diverse polysaccharides simultaneously, the concept of deconstructing synthetic polymers in the form of mixed plastics waste with advanced enzyme mixtures is an exciting research direction beyond PET to other polyesters, natural fibers (e.g., cellulose from cotton, proteins from wool) (70), polyamides, polyurethanes (71), and other polymers susceptible to enzymatic depolymerization. Going forward, the design of multienzyme systems for depolymerization of mixed polymer wastes is a promising and fruitful area for continued investigation.

## Methods

### Plasmid Construction.

pET21b(+)-based expression plasmids for *I. sakaiensis* genes, homologous genes, and mutants were generated as further described in Dataset S1.

### Protein Expression and Purification.

*E. coli*-based protein expression and chromatographic purification is described in *SI Appendix*, *Supplementary Materials and Methods*.

### Crystallization and Structure Determination.

MHETase was crystallized in four conditions, including a seleno-methionine–labeled version for single-wavelength anomalous diffraction phasing. All X-ray data collections were performed at Beamline I03 at the Diamond Light Source. Detailed methods and statistics are provided in *SI Appendix*, *Supplementary Materials and Methods* and Table S1**.**

### Molecular Simulations.

MD simulations were performed for solvated MHETase both in the free state and with MHET bound at the active site. All systems were built in CHARMM (39) and simulations utilized the CHARMM forcefield (41). Classic MD simulations were run with NAMD (40); QM/MM simulations, including 2D umbrella sampling free-energy calculations, were run in Amber (42, 72). Additional simulation details are in *SI Appendix*, *Supplementary Materials and Methods*.

### Bioinformatics.

A total of 6,671 tannase family sequences were retrieved via PSI-BLAST against the NCBI nonredundant database (50). Phylogenetic analyses were conducted with MEGA7 (73). Additional details are in *SI Appendix*, *Supplementary Materials and Methods*.

### MHETase Kinetics and Turnover Experiments.

MHETase and mutant enzymes were incubated with MHET, MHEI, or MHEF and reactions quenched with methanol and a heat treatment at 85 °C for 10 min. Hydrolysis extent was measured by HPLC as described in *SI Appendix*, *Supplementary Materials and Methods* and Table S6.

### Molecular Docking.

MHET, MHEI, and MHEF docking into MHETase were modeled and prepared using tools in Schrödinger. Substrate docking simulations were conducted using Induced Fit Docking simulations in Schrödinger as described in *SI Appendix*, *Supplementary Materials and Methods*.

### Ligand Synthesis.

MHET, MHEI, and MHEF were prepared via the coupling and subsequent deprotection of a monotBoc-protected EG with the respective acyl chlorides as further described in *SI Appendix*, *Supplementary Materials and Methods* and Figs. S12–S14.

### MHETase Synergy with PETase.

The effect of MHETase loading and PETase loading on amorphous PET film after 96 h was measured as total product release (MHET, BHET, and TPA) via HPLC, as described in *SI Appendix*, *Supplementary Materials and Methods*.

### MHETase-PETase Chimeras.

Chimeric constructs covalently linking MHETase to PETase were generated and incubated with either MHET or amorphous PET film as described in *SI Appendix*, *Supplementary Materials and Methods*.

## Supplementary Material

Supplementary File

Supplementary File

Supplementary File

Supplementary File

Supplementary File

## Data Availability

The atomic coordinates and structure factors have been deposited in the Protein Data Bank, https://www.wwpdb.org/ (PDB ID codes 6QZ1, 6QZ2, 6QZ3, and 6QZ4).
