# Supplementary material for: Characterization and engineering of a two-enzyme system for plastics depolymerization
Source: Proc Natl Acad Sci U S A. 2020 Sep 28;117(41):25476–85. doi: 10.1073/pnas.2006753117 (PMC7568301; doi:10.1073/pnas.2006753117)
Supplement: Supplementary File [file pnas.2006753117.sapp.pdf]

Supporting Information for:

Characterization and engineering of a two-enzyme system for plastics depolymerization

Brandon C. Knott[a,1], Erika Erickson[a,1], Mark D. Allen[b,1], Japheth E. Gado[a,c,1], Rosie Graham[b], Fiona L. Kearns[d], Isabel Pardo[a], Ece Topuzlu[a,e], Jared J. Anderson[a], Harry P. Austin[b], Graham Dominick[a], Christopher W. Johnson[a], Nicholas A. Rorrer[a], Caralyn J. Szostkiewicz[a], Valérie Copié[e], Christina M. Payne[c], H. Lee Woodcock[d], Bryon S. Donohoe[f], Gregg T. Beckham[a,2], and John E. McGeehan[b,2]

a. Renewable Resources and Enabling Sciences Center, National Renewable Energy Laboratory, Golden CO, USA
b. Centre for Enzyme Innovation, School of Biological Sciences, Institute of Biological and Biomedical Sciences, University of Portsmouth, UK
c. Department of Chemical and Materials Engineering, University of Kentucky, Lexington KY, USA
d. Department of Chemistry, University of South Florida, Tampa FL, USA
e. Department of Chemistry and Biochemistry, Montana State University, Bozeman MT, USA
f. Biosciences Center, National Renewable Energy Laboratory, Golden CO, USA

[1]These authors contributed equally to this work
[2]Email: john.mcgeehan@port.ac.uk; gregg.beckham@nrel.gov

Table of Contents

Supplementary Materials and Methods

Supplementary Figures
Fig. S1 Sequence alignment and structural homology
Fig. S2 Alternate positions of residue Phe415 captured in multiple crystal structures
Fig. S3 Effect of calcium binding on MHETase motion from molecular dynamics simulations
Fig. S4 Molecular dynamics of MHET binding at MHETase active site
Fig. S5 Time-traces for key distances in MD simulations of MHET bound at MHETase active site
Fig. S6 One-dimensional potentials of mean force (PMF) for acylation and deacylation steps
Fig. S7 Post-acylation simulation of active site and reaction products
Fig. S8 Conservation analysis of 6,671 tannase family sequences
Fig. S9 Amino acid frequencies of active-site positions in MHETase within 6 Å of the MHET substrate
Fig. S10 Disulfide bond cysteines in 6,671 tannase family sequences
Fig. S11 Phylogenetic analysis of 120 tannase family sequences
Fig. S12 Chemical structure and validation by NMR of synthesized mono-(2-hydroxyethyl)-isophthalate
Fig. S13 Chemical structure and validation by NMR of synthesized mono-(2-hydroxyethyl)-furanoate
Fig. S14 Chemical structure and validation by NMR of synthesized mono-(2-hydroxytheyl)-terephthalate
Fig. S15 Flexible molecular docking studies for MHET, MHEF, and MHEI
Fig. S16 SEM of amorphous PET film after 96 h enzyme treatment
Fig. S17 Evolutionary analysis by Maximum Likelihood method of known and putative TPA gene clusters
Fig. S18 Schematic representation of putative TPA catabolic gene clusters
Fig. S19 Sequence identity matrices for putative TPA catabolic proteins obtained from pairwise alignments

Supplementary Tables
Table S1 Crystallographic data, model refinement, and crystallization conditions of *Is*-MHETase
Table S2 Tannase family sequences used in phylogenetic analysis
Table S3 Michaelis-Menten kinetic parameters
Table S4 Synergistic degradation of amorphous PET film
Table S5 Putative protocatechuate-dioxygenases in *Hydrogenophaga* sp. PML113 and *Comamonas thiooxydans*
Table S6 Summary of conditions tested for quenching MHETase enzymatic activity

Captions to Supplementary Movies
Movie S1 MHETase acylation reaction
Movie S2 Release of acylation product ethylene glycol
Movie S3 MHETase deacylation reaction

Supplementary Data (Excel file)
Table SD1 Plasmid construction
Table SD2 Synthesized DNA fragments
Table SD3 Primers

Supplementary References

www.pnas.org/cgi/doi/10.1073/pnas.2006753117

Supplementary Materials and Methods

Plasmid construction

pET-21b(+) (EMD Millipore)-based plasmids for expression of the various *Ideonella sakaiensis* PETase and MHETase enzymes, as well as homologous, and mutant proteins were either synthesized by Twist Bioscience or constructed using NEBuilder® HiFi DNA Assembly Master Mix (New England Biolabs) and/or the Q5® Site-Directed Mutagenesis Kit (New England Biolabs) such that each protein has a C-terminal hexa-histidine epitope tag. For DNA assembly, DNA fragments were either amplified using Q5® High-Fidelity 2X Master Mix (New England Biolabs) or synthesized by Integrated DNA Technologies. Kits and master mixes were used according to the manufacturer's instructions. Plasmids were initially transformed into NEB® 5-alpha F'I^q Competent *E. coli* (New England Biolabs) and confirmed using Sanger sequencing by GENEWIZ, Inc. Specific strain construction details and sequences of synthesized DNA and PCR primers are provided in the ***Supplementary Data*** file.

Protein expression and purification

For initial screening for soluble protein expression of the proteins of interest, various cell lines and induction methods (1) were used to purify protein for kinetic assays. For expression and purification, OverExpress™ *E. coli* C41 (DE3) (Lucigen) cells were transformed with pET21b(+) plasmid constructed with the gene of interest (***Supplementary Data*** file). Single colonies from transformation were then inoculated into a starter culture of Luria Broth (LB) media containing 100 µg/mL ampicillin and grown at 37°C overnight. The starter culture was inoculated at a 100- fold dilution into a 2xYT medium containing 100 µg/mL ampicillin and grown at 37°C until the optical density measured at 600 nM (OD600) reached 0.6-0.8. Protein expression was then induced by addition of isopropyl β-D-1-thiogalactopyranoside (IPTG) to a final concentration of 1 mM. Cells were maintained at 20°C for 18 to 24 hours following IPTG induction, harvested by centrifugation, and stored at -80°C until purification. Harvested cells were resuspended in a lysis buffer (300 mM NaCl, 10 mM imidazole, 20 mM Tris HCl, pH 8.0,) and lysed using a bead beater (BioSpec Products, Inc.). Lysate was clarified by centrifugation at 40,000 x *g* for 45 minutes. Clarified lysate was then applied to a 5 mL HisTrap HP (GE Healthcare) Ni-NTA column using an ÄKTA Pure chromatography system (GE Healthcare) and eluted using 300 mM NaCl, 300 mM imidazole, 20 mM Tris HCl, pH 8.0. Resulting fractions containing proteins of interest were applied to a Sephacryl S-100 26/60 HR (GE Healthcare) size exclusion column equilibrated with 100 mM NaCl, 20 mM Tris HCl, pH 7.5 for biochemical assays, or the fractions were applied to a Superdex 75 pg 16/60 (GE Healthcare) size exclusion column equilibrated with 100 mM NaCl, 20 mM Tris HCl, pH 7.5 for crystallography. Protein in eluted fractions from Ni-NTA and size exclusion columns were assessed using SDS-PAGE with Coomassie staining and Western blot using primary antibody against the hexa-histidine epitope tag (Invitrogen). Total protein was assessed by BCA assay (2). For proteins that did not express, or expressed in inclusion bodies, using the above described expression protocol, additional *E. coli* expression cell lines were tested, including Rosetta 2 (DE3) (Novagen), BL21 (DE3), and Lemo21 (DE3) (New England Biolabs), as was expression by autoinduction at 30°C in ZYP-5052 media (1). All studies reported were performed with freshly purified protein. The use of lyophilized protein was attempted, however, specifically for MHETase, inconsistent enzyme inhibition behaviors were observed.

MHETase:PETase chimeras. Chimera proteins were expressed and purified as described above with the following noted exceptions: Single colonies from transformation into C41 (DE3) competent cells were used to inoculate a starter culture of 200 mL Terrific Broth (TB) media containing 100 µg/mL ampicillin for overnight outgrowth at 37°C. From the starter culture, 50 mL was used to inoculate 1 L of TB media containing 100 µg/mL ampicillin. For purification, cells were disrupted by sonication. In the final chromatography step a Superdex 200 pg 16/600 (GE Healthcare) size exclusion column equilibrated with 100 mM NaCl, 20 mM Tris HCl, pH 7.5 was used.

Crystallography

After purification, as described above, MHETase protein was concentrated to a range of concentrations (9-14 mg/mL) and dialyzed into 100 mM NaCl, 10 mM Tris, pH 7.0 for crystallography.

For seleno-methionine labeling of MHETase, K-MOPS minimal media was used (3). Cells were grown to an OD_{600} of 0.5 after which 100 mg/L of DL-seleno-methionine (Sigma), 100 mg/L lysine, threonine and phenylalanine, leucine, isoleucine and valine were added as solids. IPTG (1 mM final concentration) was then added after 20 min and cells were grown for a further 16 h at 20°C. Seleno-methionine labeled protein was purified as described above. MHETase was crystallized at a range of concentrations from 9-14 mg/mL by sitting-drop vapor diffusion. Several conditions yielded crystals, four of which were used to obtain datasets, one of which contained seleno-methionine labelled protein.

The crystals were cryo-cooled in liquid nitrogen after the addition of glycerol to 20% (v/v) while leaving the other components of the mother liquor at the same concentration. Seleno-methionine MHETase crystals belonging to space group $P22_12_1$ were used to obtain phase information using the I03 beamline at the Diamond Light Source (Oxford, UK). Data were obtained from 3600 images collected at 0.9795 Å with 0.1° increments. All images were integrated using XDS (4) and scaled using SCALA (5). Phases were obtained using PHASERSAD in the CCP4i software in combination with PARROT and SHELXD (6, 7). The initial output was subsequently built using BUCCANEER and further refined using iterative rounds of COOT and PHENIX (8-10). One molecule of MHETase was observed in the asymmetric unit of the $P22_12_1$ seleno-methionine SAD dataset. Three additional native datasets, each containing 1800 images collected at 0.1° increments, were collected at beamline I03 of the Diamond Light Source. The structure of native MHETase were obtained using molecular replacement from a refined molecule of MHETase obtained initially from the seleno-methionine SAD data. All structures were refined using iterative rounds of COOT and PHENIX (8-10). Cell constants, crystallographic data and details of the refined models are summarized in **Supplementary Table S1** (11). Structural figures were generated with PYMOL (Schrödinger, LLC) with accompanying sequence alignments generated in Clustal W (12) and rendered using ESPript 3.0 (13).

Ligand synthesis

MHET, MHEI, and MHEF synthesis. Mono(2-hydroxyethyl) terephthalate (MHET) and mono(2-hydroxyethyl) isophthalate (MHEI) and mono(2-hydroxyethyl) furanoate (MHEF) were synthesized via the condensation of either terephthloyl chloride, isophthloyl chloride, or the acyl chloride of 2,5-furan dicarboxylic acid, respectively, with monoprotected ethylene glycol (tBOC-EG) which was subsequently deprotected to yield the final product.

Initially, tBOC-EG was prepared by stirring one molar equivalent of ethylene glycol (EG) with one molar equivalent of di-tert-butyl decarbonate with 0.01 equivalents of 4-dimethylaminopyridine (DMAP) as a catalyst in dichloromethane (DCM). The reaction mixture was allowed to stir for 24 hours and was subsequently washed with DI water, 1 M HCl, and brine follow by drying with sodium sulfate. The solvent was removed, and the product was purified via silica gel column chromatography to yield the mono-protected tBOC-EG. The yield of this reaction was 60% at a final purity of 99+% (via NMR and HPLC).

To form MHET, MHEI, or MHEF either one molar equivalent of terephthloyl chloride, isophthloyl chloride, or the acyl chloride of 2,5-furan dicarboxylic acid, respectively, was dissolved in DCM with one molar equivalent of tBOC-EG. One molar equivalent of triethylamine (TEA) was then added in dropwise over a period of 30 minutes. The reaction solution was subsequently washed with DI water and brine and then dried over sodium sulfate prior to removing the DCM. The crude product was subsequently taken up in a mixture of 10% acetone in DCM and purified via silica gel chromatography.

NMR for MHET, MHEF, and MHEI is provided in **Supplementary Figures S18, S19,** and **S20,** respectively.

Enzyme activity and synergy with PETase

Quenching enzymatic reactions. Previous studies of MHETase activity report the use of an equal volume of pH 2.5 sodium phosphate buffer and a heat treatment at 80-85°C for 10 min to quench enzymatic activity (14, 15). We found this to be an inconsistent method of quenching enzyme activity, such that some level of enzyme activity continues after treatment, as quantified by HPLC analysis. To determine a reliable method for quenching enzymatic activity we performed quenching trial experiments in triplicate for reactions containing 250 µM MHET, 90 mM NaCl, 10% (v/v) DMSO, 45 mM sodium phosphate, pH 7.5, at 30°C, for both reactions containing enzyme in order to compare the quenching capacity of a given method, and reactions without enzyme to evaluate the level of non-enzymatic hydrolysis caused by the treatment method. Quenching solution components intended to denature the enzyme, such as a reducing agent (TCEP), chaotropic agent (GuHCl), or strong acid (6N HCl) proved either inadequate to completely quench enzymatic activity, or rather resulted in high levels of acid-mediated hydrolysis of the substrate. The active-site inhibitor PMSF inconsistently quenched enzymatic activity. Polar solvents (ethanol, methanol, and Isopropanol) were most effective at quenching enzymatic activity. The quenching solutions used are summarized in **Supplementary Table S6**. Based on the results, an equal volume of 100% methanol followed by a heat treatment at 85°C for 10 min was selected as the most reliable method of quenching, which also yields the lowest levels of non-enzymatic hydrolysis of MHET.

Determination of enzyme turnover rates. Comparative assays for each enzyme were performed at the same enzyme and substrate concentration. Reactions were performed in triplicate over a 15 min time course using 5 nM enzyme concentration and 250 µM MHET in 90 mM NaCl, 10% (v/v) DMSO, 45 mM sodium phosphate, pH 7.5, at 30°C. Reactions were terminated using an equal volume of 100% methanol followed by heat treatment at 85°C for 10 min. Product and substrate were quantified by HPLC. Apparent turnover rate (k_{cat}) was determined by terephthalic acid (TPA) produced.

Michaelis-Menten kinetics of MHETase and variants. Reactions were performed in triplicate over a 10 min time course using 5 nM enzyme and substrate concentrations ranging from 10 µM to 250 µM MHET in 90 mM NaCl, 10% (v/v) DMSO, 45 mM sodium phosphate, pH 7.5, at 30°C. Each reaction was terminated using an equal volume of 100% methanol and heat treatment at 85°C for 10 min. Product and substrate were quantified by HPLC. Initial reaction velocities were calculated from TPA produced over time and kinetic parameters were determined by nonlinear regression of the initial velocities fit to the Michaelis-Menten equation. The wild-type MHETase and both homologous enzymes were fitted to the Michaelis-Menten model with substrate inhibition (Eq. 1) while the MHETase S131G mutant was fitted to the simple Michaelis-Menten model (Eq. 2) using GraphPad Prism version 8.4.1 for MacOS (GraphPad Software, San Diego, California USA), as follows:

$$v = \frac{V_{max}\,[S]}{K_m + [S](1 + \frac{[S]}{K_i})} \quad \text{(Eq. 1)}$$

$$v = \frac{V_{max}\,[S]}{K_m + [S]} \quad \text{(Eq. 2)}$$

While both substrate inhibition and product inhibition are possible in these reactions, the relationship between initial reaction velocity and initial substrate concentration indicates substrate inhibition predominates in these reaction conditions. Low substrate concentrations were considered in these kinetic studies in order to minimize the effect of substrate inhibition.

Enzymatic degradation of PET film. Amorphous PET film (2-3% crystallinity, Goodfellow, UK) was incubated with enzyme of interest in polypropylene tubes containing 90 mM NaCl, 10% (v/v) DMSO, 45 mM sodium phosphate, pH 7.5, at 30°C for 96 hours. The reaction was terminated by addition of equal volume 100% methanol and PET coupons were removed from the reaction solution. The reaction solution was heat treated at 85°C for 10 minutes. PET coupons were washed with consecutive rinses of 1% SDS, 100% DMSO, ultrapure water, and 95% ethanol. Coupons were then vacuum dried for 24 h at 60°C for scanning electron microscopy.

Activity assay of MHETase with non-MHET substrates. Evaluation of MHETase activity was performed in triplicate using 5 nM enzyme concentration and 25 µM, 50 µM, and 250 µM substrate concentration at 30°C for 24 h in a 0.5 mL reaction volume. The reaction was carried out in 90 mM NaCl, 10% (v/v) DMSO, 45 mM sodium phosphate, pH 7.5, reaction buffer with three concentrations of each substrate (MHET, MHEI, or MHEF). Reactions commenced upon addition of enzyme or an equal volume of reaction buffer for the no enzyme controls. At the end of 24 h the reactions were terminated using an equal volume of 100% DMSO and heat treatment at 85°C for 10 min. Product and substrate were analyzed by HPLC. Values reported as percentage of substrate hydrolyzed into product.

HPLC method. Standards of BHET, TPA, 2,5-furandicarboxylic acid, and isophthalate were obtained from Sigma Aldrich. MHET, MHEI, and MHEF were synthesized as described above. Analyte analysis of samples was performed on an Agilent 1260 LC system (Agilent Technologies, Santa Clara, CA) equipped with a G1315A diode array detector (DAD). Each sample and standard were injected using a volume of 10 µL onto a Phenomenex Luna C18(2) column, 5 µm, 4.6 x 150 mm (Phenomenex, Torrance, CA). The column temperature was maintained at 40°C and the mobile phase used to separate the analytes of interest was composed of 20 mM phosphoric acid in water (A) and 100% methanol (B). The separation was carried out using a constant flow rate of 0.6 mL/min and a gradient program of: at t = 0 min (A) = 80% and (B) = 20%; at t = 15 min (A) = 35% and (B) = 65%; at t = 15.01 min through 20 min (A) = 80% and (B) = 20% for a total run time of 20 min. The calibration curve for each analyte was evaluated between concentrations of 0.1 – 200 mg/L. DAD detection at a wavelength of 240 nm was performed for each analyte. Ten calibration standards were used with an r^2 coefficient of 0.995 or better and a calibration verification standard (CVS) at 100 mg/L for each analyte was analyzed every 18 samples to ensure the integrity of the initial calibration. Samples were diluted with an equal volume of ultrapure water for analysis.

Scanning Electron Microscopy.

Dried PET coupons sized 2.5 cm x 0.5 cm were placed on aluminum stubs using carbon tape, and were sputter coated with 9 nm of iridium. SEM imaging was performed using an FEI quanta 400 FEG instrument under low vacuum (0.45 torr), beam-accelerating voltage of 25 keV.

Bioinformatics

Sequence selection and conservation analysis. 6,671 tannase family sequences were retrieved by a PSI-BLAST search against the NCBI non-redundant database with *Is* MHETase (A0A0K8P8E7.1) as initial query sequence on November 16, 2018. A total of three iterations of the PSI-BLAST search were carried out, and all 6,671 hits had E-values of 1e-50 or better. A multiple sequence alignment of the 6,671 tannase family sequences was carried out with MAFFT (16). The amino acid conservation at each site of the multiple sequence alignment was evaluated by computing the relative entropy according to the following equation (17):

$$\mathrm{R.E} = \sum_{i=1}^{20}\left(p_i \log\frac{p_i}{p_i^{MSA}}\right) \quad \text{(Eq. 3)}$$

where p_i is the frequency of the ith amino acid in the given site and p_i^{MSA} is the overall frequency of the ith amino acid in the multiple sequence alignment.

Phylogenetic analysis. Through a keyword search of the NCBI protein database (https://www.ncbi.nlm.nih.gov/protein) with BioPython (18), functional annotation for the 6,671 sequences was retrieved. From the sequence description in the NCBI database, 338 and 51 sequences of the 6,671 sequences were clearly annotated as ferulic acid esterases, or as tannases, respectively. Profile hidden Markov models (HMMs) were constructed for ferulic acid esterases and tannases with the dataset of 338 and 51 sequences, respectively, using the HMMER software (version 3.1b2) (19). Sequence identity thresholds of 95% and 60% were, respectively, applied to the set of 338 ferulic acid esterases and 51 tannases resulting in a set of 120 sequences (86 FAEs, 31 tannases, *Is*-MHETase, and 2 *Is*-MHETase close homologs). The 120 sequences were aligned with MAFFT (16) and phylogenetic analysis with 1000 bootstrap replicates was conducted in MEGA7 (20). For the phylogenetic tree, the evolutionary distances were computed using the JTT matrix-based method (21). The minimum evolution tree was searched using the Close-Neighbor-Interchange (CNI) algorithm (22) at a search level of 1, and the Neighbor-joining algorithm (23) was used to generate the initial tree. Gaps in the alignment were handled using pairwise deletion. There were a total of 1440 positions in the final dataset.

Identification of homologous enzymes. MHETase shares low sequence similarity (<53%) with most sequences in the tannase family, with the exception of two homologous sequences, one from *Comamonas thiooxydans* (strain: NCBI:txid363952, protein: Genbank WP_080747404.1) (24) and one from *Hydrogenophaga* sp. PML113 (strain: NCBI:txid1899350, protein:Genbank WP_083293388.1). In the time since identification of this *C. thiooxydans* sequence, this accession entry was removed from Genbank upon request of the submitter. Three other strains of *C. thiooxydans* also carry this sequence (protein:Genbank WP_034389536.1), though lacking 28 residues at the N-terminus. These include *C. thiooxydans* strains DS1 (protein:INSDC KGH18114.1), DF1 (protein:INSDC KGH28153.1), and DF2 (protein:INSDC DGH05124.1). Using the original protein accession sequence (WP_080747404.1), SignalP prediction indicates the sequence encodes a 70-residue signal sequence. We attempted expression of both *C. thiooxydans* and *Hydrogenophaga* sp. PML113 enzymes without the predicted signal peptide, however the enzymes did not express.

Molecular docking

MHETase structure preparation. MHETase structure was taken from starting structures used for molecular dynamics simulations. The MHETase structure was prepared with Schrodinger's Protein Preparation Wizard in Schrodinger) (24-26). PropKa was used to optimize hydrogen bonds at pH 7.0; OPLS3 force field (27) was used to conduct a restrained minimization on all heavy atoms (to ensure less than 0.30 Angstrom deviation from starting structure position).

Ligand structure preparation. MHET and MHEI structures were built in Schrodinger using Maestro Workspace tools. All ligands were energetically minimized using Schrodinger LigPrep (28) according to OPLS3 force field (29). Ionization states of MHET and MHEI were requested with Epik at pH of 7.0 (30, 31) although no additional ionization states were generated.

Flexible ligand/flexible receptor docking. Induced Fit Docking (IFD) is Schrodinger's flexible ligand/flexible receptor docking tool (28, 30, 32-34). IFD utilizes two other Schrodinger modules, Prime for amino-acid side chain prediction and refinement, and Glide for ligand docking, to achieve binding site flexibility during docking simulations. Ligands were docked into MHETase active site (determined by co-crystallization with benzoic acid) by trimming (mutating and back-mutating) all residues within 5 Å of the catalytic triad, except for the catalytic triad. This was necessary as attempts to mutate catalytic triad residues to Alanine then back-mutate after initial docking (as is procedure in IFD) would result in chemically incompetent catalytic triad residues. After docking and amino-acid refinement, binding modes were scored and ranked using the Glide XP scoring function. Resultant predicted binding poses were then analyzed to determine if each pose would result in cleavage of an ester bond, such poses were determined to be chemically relevant. Those chemically relevant poses with the lowest predicted binding free energies (i.e. lowest Glide XP score) are discussed in detail in the Results.

Molecular simulations

The starting point for molecular dynamics (MD) simulations was chain A of the 1.8 Å resolution structure (PDB code 6QZ4). This structure was chosen because it has electron density for the widest range of residues (6QZ3 lacks residues 36-39 and 6QZ1 lacks residues 56-60). The bound calcium ion and the crystal waters are maintained (sulfate is deleted). For a variety of residues with alternate conformations, conformation A was chosen for the following: Ser143, Ile149, Ser240, and Asn403. Conformation B was chosen for Ser401 and Leu486. Initial proposal for protonation states was given by H++ server (http://biophysics.cs.vt.edu/H++) (35) at pH 7.0, consistent with Yoshida *et al.* (15) reaction conditions. Of the acidic residues (glutamic and aspartic acid), Glu230 was determined to be protonated. For histidine residues, His91 and His528 are singly protonated at ND1, His293, His 467, and His488 are singly protonated at NE2, and His166 and His241 are doubly protonated. The overall charge on MHETase with these protonation states is -6; 6 sodium ions were added to the solution phase to neutralize. Five disulfides are formed: Cys51 - Cys92, Cys224 - Cys529, Cys303 - Cys320, Cys340 - Cys348, and Cys577 - Cys599.

All simulations were built using CHARMM (36) version 43a1 and simulated with the CHARMM36 force field (37) for the protein, CHARMM force field for carbohydrates (38, 39), and TIP3P water molecules (40). Topologies and forcefield parameters for MHET were generated by CGenFF program version 2.2.0 (41, 42) for use with CGenFF forcefield version 4.0 (43, 44).

The simulation box is cubic, with each box side approximately 110 Å long. Approximately 132,000 atoms are modeled in each system. Classical MD simulations of 150 ns in length were run in triplicate for the following five scenarios: 1), 2) free MHETase (no substrate bound) with calcium ion bound at calcium binding site (Phe415 open and closed), 3), 4) MHETase with MHET bound at active site with calcium ion bound at calcium binding site (Phe415 open and closed), and 5) free MHETase with neither substrate nor calcium ion bound (Phe415 open). For the simulations with bound MHET, the initial state was prepared as follows. Near neutral pH, MHET exists in solution as a salt, thus the carboxylate moiety of MHET is deprotonated in our simulations (for reference, the pKa of the first and second acidic moieties of TPA are 3.54 and 4.46 at 25°C (PubChem)). The initial configuration for MHET bound at

the active site of MHETase was prepared in the following manner. *Is* PETase bound with PET tetramer from a prior molecular docking study (45) was trimmed back to a hydroxyethyl-capped PET dimer maintaining the ester bond nearest to the catalytic triad as well as the repeat units on either side. Following MM and QM/MM minimization**,** restraints were placed on two distances in order to prepare an enzyme-substrate configuration primed for catalysis: the nucleophilic attack distance between Ser225 oxygen and PET carbon (target: 2.0 Å), and the scissile C-O ester bond distance (target: 1.4 Å). Force constants of 200 kcal/mol/$Å^2$ were utilized in both cases. The catalytic residues of MHETase were then aligned with those of PETase. Subsequently trimming the PET dimer back to the heavy atoms it shares in common with MHET gave the starting point for MD simulations with MHET bound.

All classical MD simulations were performed at 303 K to match the conditions for hydrolytic assays performed by Yoshida *et al.* (15). Systems were density equilibrated for 1 ns at a constant pressure of 1 atmosphere and constant temperature of 303 K (controlled via the Nosé-Hoover barostat and thermostat); subsequent production runs were performed with constant volume and temperature (303 K) in NAMD 2.9 (46). All bonded hydrogen distances were constrained utilizing the SHAKE algorithm (47). The timestep was 2 fs. A nonbonded cutoff distance of 10 Å was utilized, with a switching distance of 9 Å, and a nonbonded pair list distance of 13 Å. The long-range electrostatics were described via the Particle Mesh Ewald (PME) method with a sixth order b-spline, a Gaussian distribution with a width of 0.312 Å, and 1 Å grid spacing. The velocity Verlet multiple timestepping integration scheme was used, with the full nonbonded interactions evaluated every timestep, full electrostatics interactions evaluated every 3 timesteps, and 6 timesteps between atom reassignments.

Following 1 ns of dynamics with classical forcefield, the CHAMBER utility (48) of ParmEd version 3.0.3 was used to convert the CHARMM coordinate, topology, parameter, and protein structure files to AMBER formatted coordinate and topology files for hybrid quantum mechanics/molecular mechanics (QM/MM) simulations by the sander program of AMBER version 12 (49). The AMBER software was used to carry out all QM/MM calculations (50, 51) with the Self-Consistent Charge Density-Functional Tight-Binding (SCC-DFTB) semiempirical QM method using the Third-Order Parameterization for Organic and Biological Systems (3OB) to describe the QM region (52). An 8 Å cutoff was used for nonbonded interactions and PME used for long-range electrostatics. Periodic boundary conditions were utilized, the timestep was 1 fs, and SHAKE was applied only to hydrogen atoms in the MM region (hydrogen atoms in the QM region were not constrained by the SHAKE algorithm). The Langevin thermostat and barostat were utilized with collision frequency of 1.0 ps^{-1} and pressure relaxation time of 2.0 ps.

The QM region includes the MHET substrate and the three catalytic residues (Ser225, Asp492, and His528, each cut across the C_α/C_β bond). For step 1 of the catalytic mechanism (acylation), there are 46 atoms in the QM region, with a QM region charge of -2. Hydrogen link atoms are utilized where covalent bonds cross the boundary between the QM and MM regions. For step 2 of the catalytic mechanism (deacylation), the ethylene glycol product is removed (as it was shown to leave the active site after the acylation step) and a single water molecule is added to the QM region, giving 39 atoms in the QM regions still with a charge of -2.

Two-dimensional free energy surfaces were prepared for acylation and deacylation via umbrella sampling simulations of each step. Order parameters utilized as reaction coordinates for both steps are the breaking and forming C-O bonds. For acylation, this is the breaking MHET ester bond ("r1") and the forming AEI bond between S125 and MHET carbonyl carbon ("r2"). For deacylation, this is the forming bond between water oxygen ("r1") and MHET carbonyl carbon and the breaking AEI bond between TPA and S125 ("r2"). Harmonic restraints are placed on these distances with force constant of 400 (kcal/mol)/$Å^2$. Windows are spaced by 0.1 Å increments for each of the two bonds (acylation: r1 between 1.3 and 3.4; r2 between 1.3 and 2.9; deacylation: r1 between 1.3 and 3.4; r2 between 1.3 and 3.9, neglecting some combinations that are particularly high energy in each case). Umbrella sampling simulations are performed in the NVE ensemble. Each window is equilibrated for 25 ps; data is collected on subsequent 500 ps. The variational free energy profile (vFEP) method (53) was utilized to produce the two-dimensional free energy profile from the probability distributions of each window. Block averaging (10 blocks for each set of umbrella sampling data) was utilized to estimate error bars on the free energy differences and reaction rate constants.

To aid in the visualization of the acylation and deacylation reactions in *Is* MHETase, QM/MM transition path sampling (TPS) simulations (54) were undertaken. In particular, the Aimless Shooting (AS) (55, 56) flavor of TPS was employed. TPS is a powerful technique for studying rare events because the trajectories that it generates are completely unrestrained and do not bias the reaction along any chosen reaction coordinate (54). The AS variety has only one adjustable parameter (dt, which here is equal to 25 fs). The simulation time for each MHETase AS trajectory is 2 ps, which was sufficient for the trajectory to relax to both stable basins for reactant and product. Other simulation parameters, including the QM region, forcefield, timestep, cutoff distances, etc. are the same as in the QM/MM two-dimensional umbrella sampling simulations, described above. Path sampling simulations were undertaken purely for illustrative purposes; no data was analyzed from these trajectories. For both acylation and deacylation, the initial configuration (which represents a configuration that is putatively part of the transition state ensemble) was taken from the end point configuration of the US window restrained to distances of 1.9 Å for both the breaking and forming C-O bonds. For acylation, 6 out of 21 trajectories were accepted (meaning they connected reactants to products). For deacylation, 13 out of 23 trajectories were accepted. For both acylation and deacylation, the movie was made from the last accepted trajectory.

Supplementary Figures

A

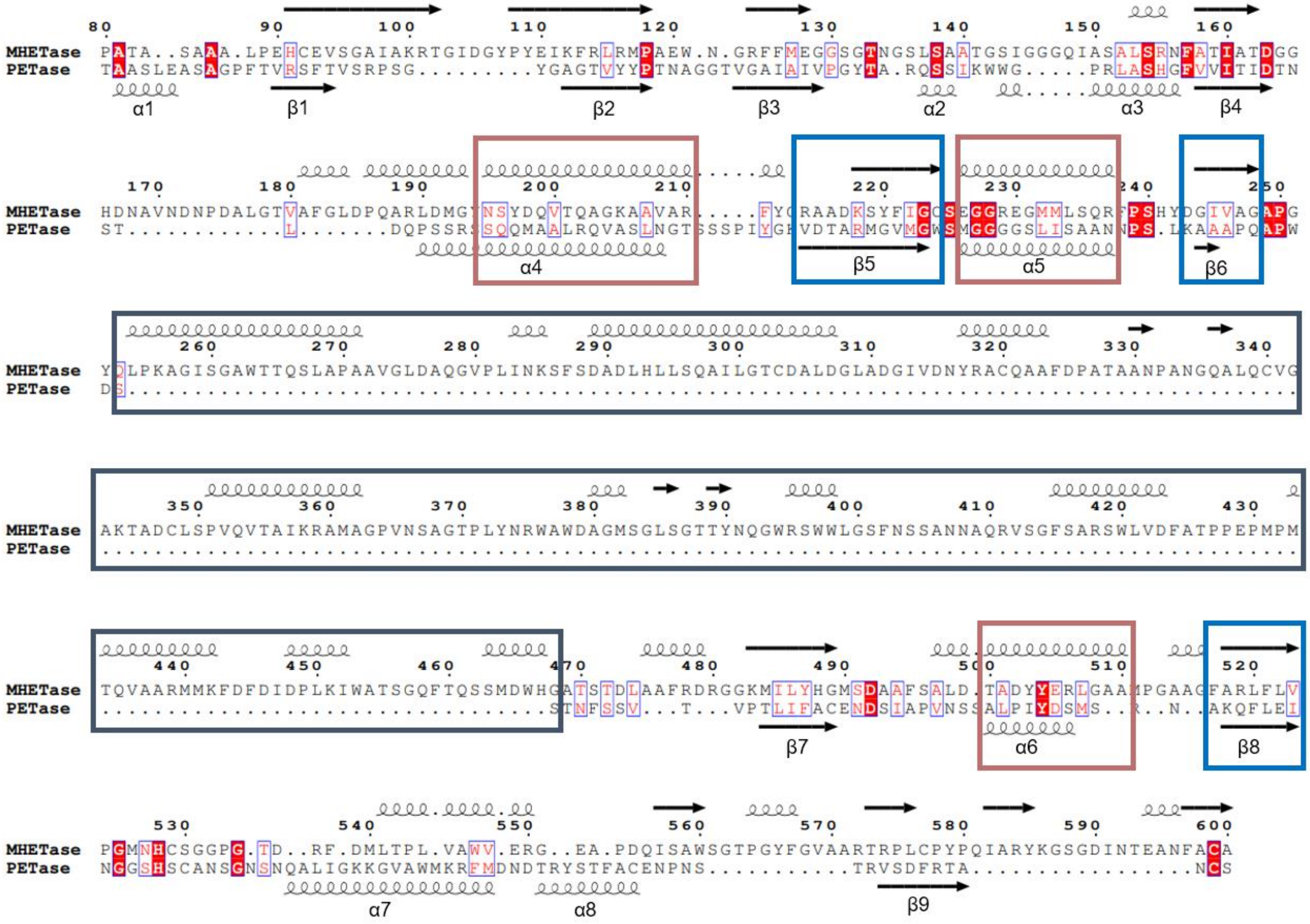

B

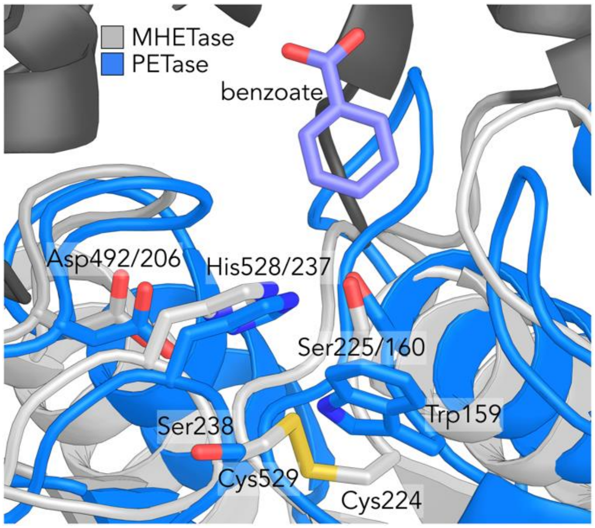

Fig. S1: A) Sequence alignment and secondary structure homology. The core sequence of *Ideonella sakaiensis* MHETase (residues 80-600) is shown aligned to PETase with regions of strong structural homology boxed in pink for common α-helices and boxed in blue for common β-strands. The lid domain is boxed in dark grey. The secondary structure elements are labelled according to the standard /β hydrolase nomenclature with α-helices depicted as spirals, β-strands as arrows, and numbering corresponding to the 3-dimensional representation presented in **Fig. 1D**. Similar residues are shown in red text with identical residues in solid red boxes. B) Active site comparison of MHETase (PDB code 6QZ3) and PETase (PDB code 6EQE). Where two residues indices are given separated by a backslash, the first number applies to MHETase and the second to PETase. Lid domain of MHETase is shown in dark gray.

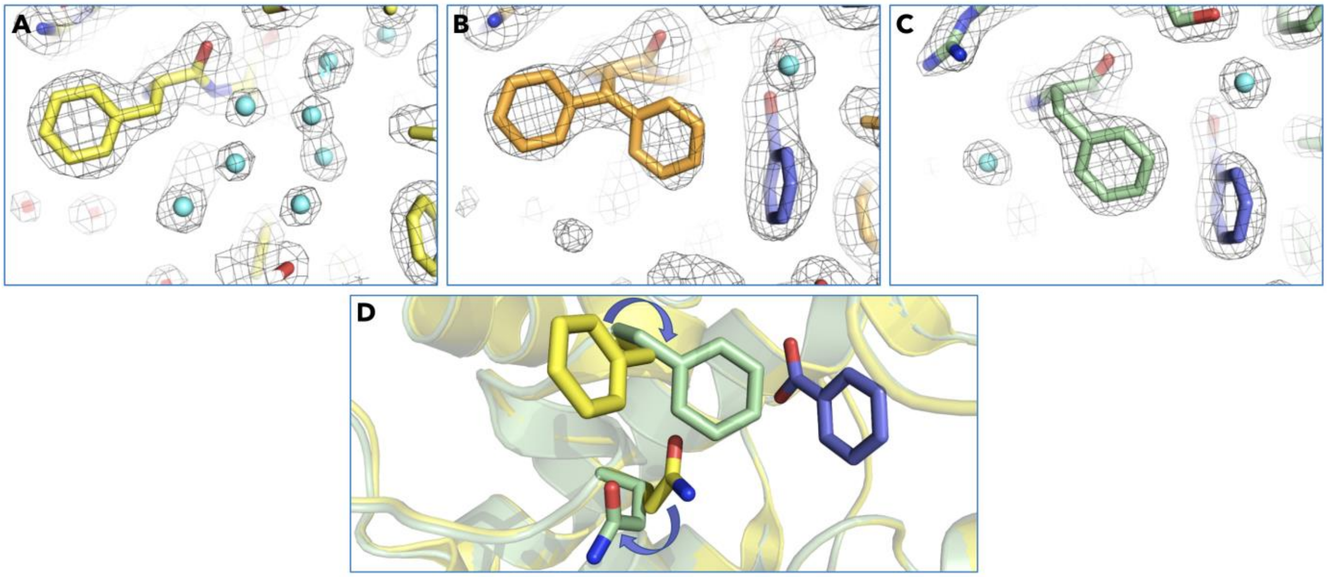

Fig. S2: Alternate positions of residue Phe415 captured in multiple crystal structures. A) The apo-structure (PDB ID: 6QZ4) with Phe415 depicted in yellow in the open conformation. The active site is populated with several water molecules (cyan spheres). The 2F_o–F_c electron density map was contoured at 1.3 σ. B) A mixed conformation of Phe451 (orange) was refined in structure PDB ID: 6QZ1. Electron density for the benzoic acid (purple) was weaker than the surrounding residues, suggesting that the site it not fully occupied; hence the alternative positions shown here likely represent a mixture of bound and free states. The 2F_o–F_c electron density map was contoured at 0.5 σ to highlight the dual conformation. C) The fully bound form of benzoic acid in the active site (PDB ID: 6QZ3) reveals Phe415 (green) in the closed conformation. The 2F_o–F_c electron density map was contoured at 1.3 σ. D) As a point of reference to **Fig. 1C**, the concerted movement of residues Gln410 and Phe415 on ligand binding is illustrated with purple arrows in a superposition of the apo enzyme (yellow) with the ligand bound state (green). The relative position of benzoic acid is depicted in purple.

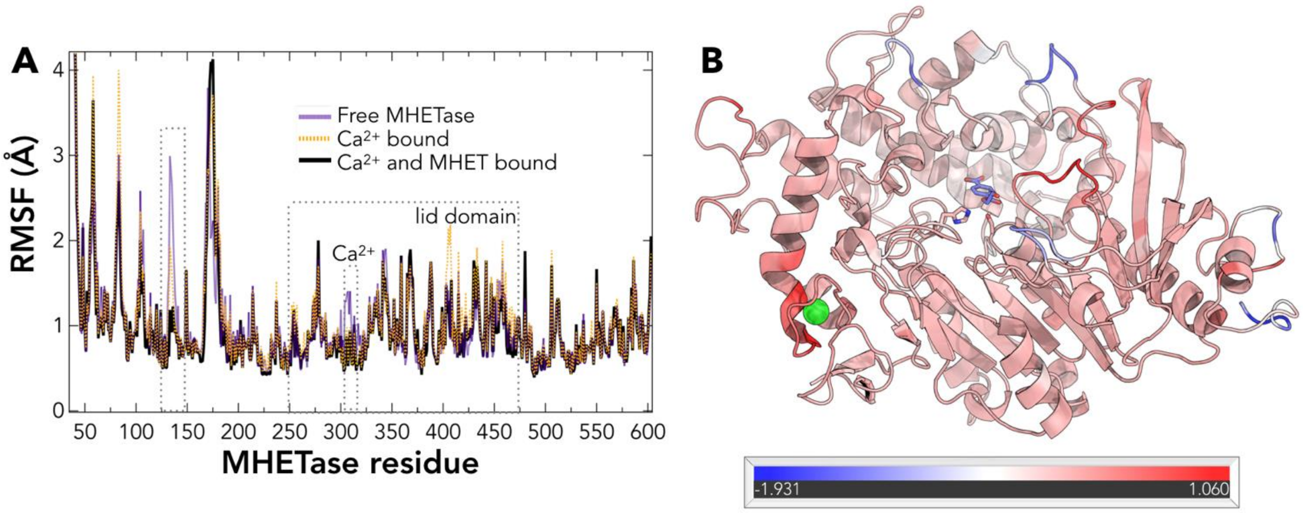

Fig. S3: Effect of calcium binding on MHETase motion from molecular dynamics simulations. A) Root mean square fluctuations (RMSF) for the heavy atoms of each MHETase amino acid (backbone and side chain atoms) for three different situations. “Free MHETase” refers to MHETase bound with *neither* calcium ion nor MHET. “Ca^{2+} bound” has calcium bound at the calcium binding site but with empty active site. “Ca^{2+} and MHET bound” has calcium bound at calcium binding site and MHET bound at the active site. Each trace represents the average RMSF from three independent MD trajectories, each of 150 ns in length. RMSF analysis was performed in CHARMM. Shown in dashed boxes are the lid domain, the region immediately surrounding the Ca^{2+} binding site, and the loop region near the active site that is significantly stabilized by Ca^{2+} binding (approximately residues 125 through 150 and appearing in red in panel B). B) MHETase structure colored by the RMSF difference between “Free MHETase” (purple trace in panel A) and “Ca^{2+} bound” (orange trace in panel A) showing the regions wherein Ca^{2+} significantly stabilizes the enzyme (red), regions where there is little effect (white/pink), and those regions where the trend is actually reversed (blue).

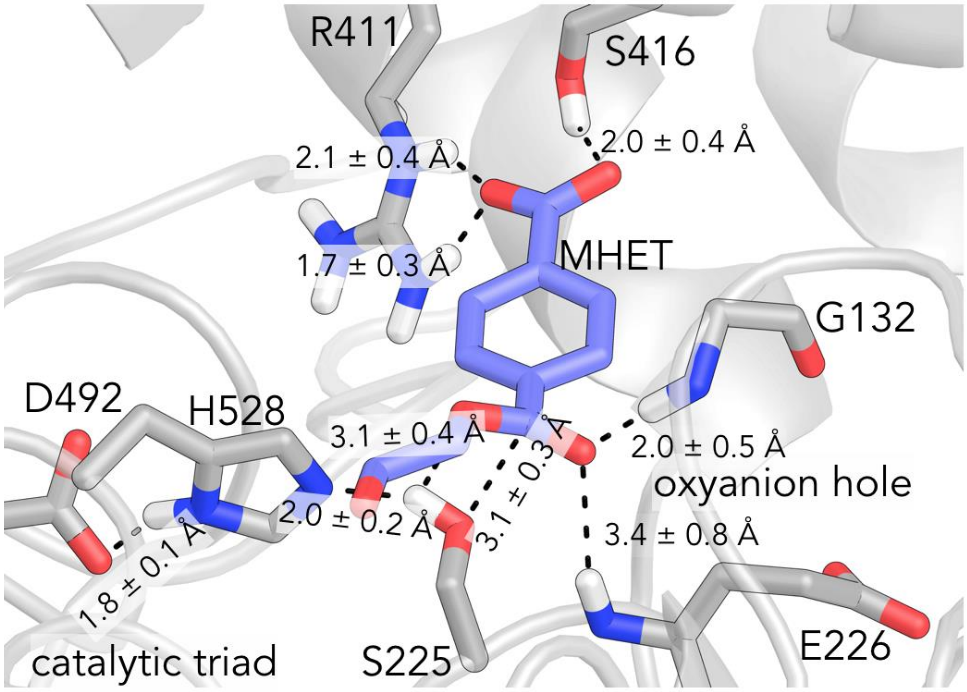

Fig. S4: Molecular dynamics of MHET binding at MHETase active site. The distances noted represent the average ± standard deviation from three independent MD simulations, each of 150 ns in length. Gly132 and Glu226 comprise the oxyanion hole and interact with the carbonyl oxygen in the Michaelis complex and throughout the acylation reaction; Arg411 and Ser416 interact strongly with the carboxylate motif.

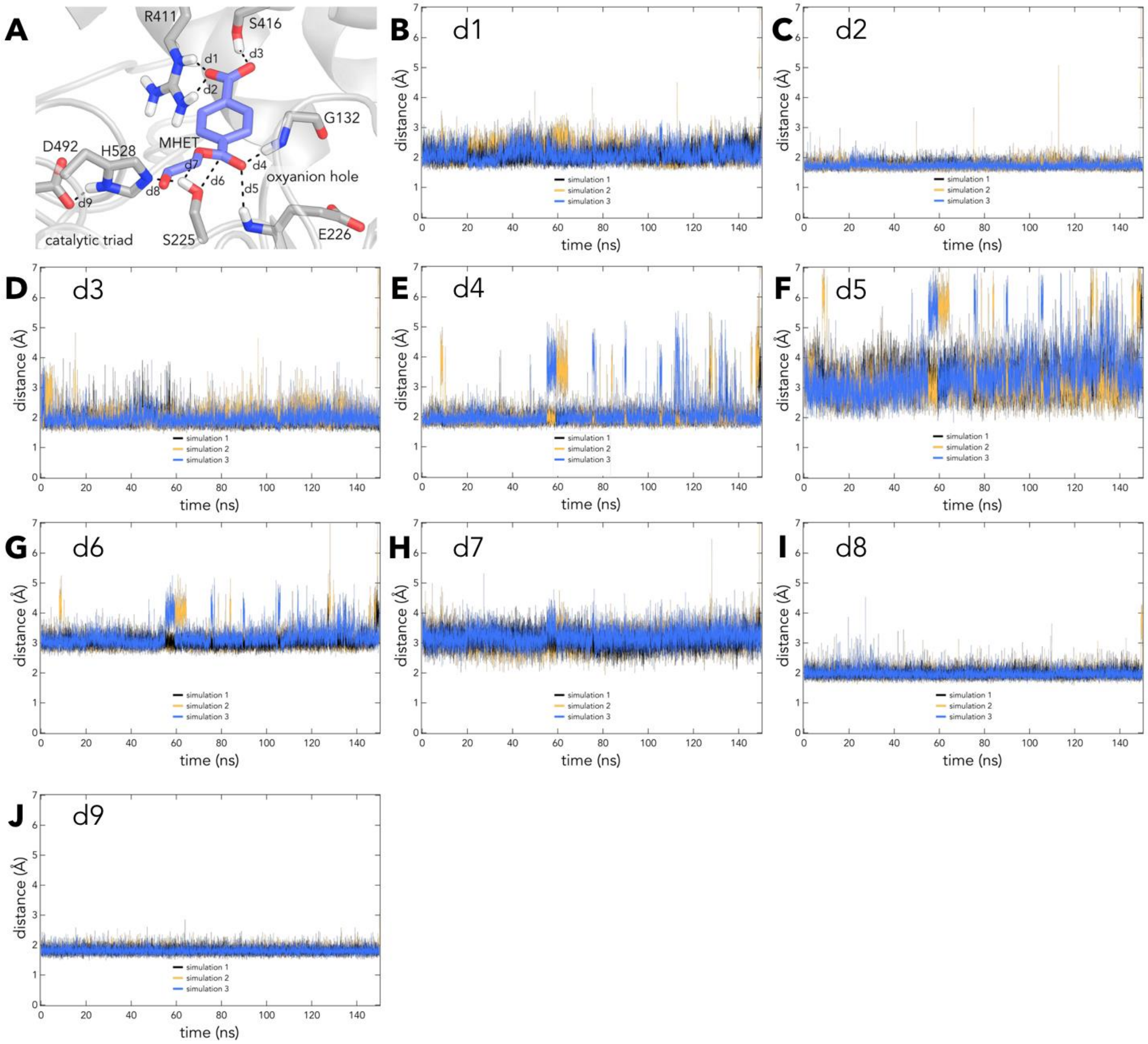

Fig. S5: Time-traces for key distances in MD simulations of MHET bound at MHETase active site. Panels B-J show the dynamic time traces for the distances annotated in panel A. These are the same distances for which averages and standard deviations are shown in Figure S4. The three simulations referenced in each panel are identical MD simulations of 150 ns in length.

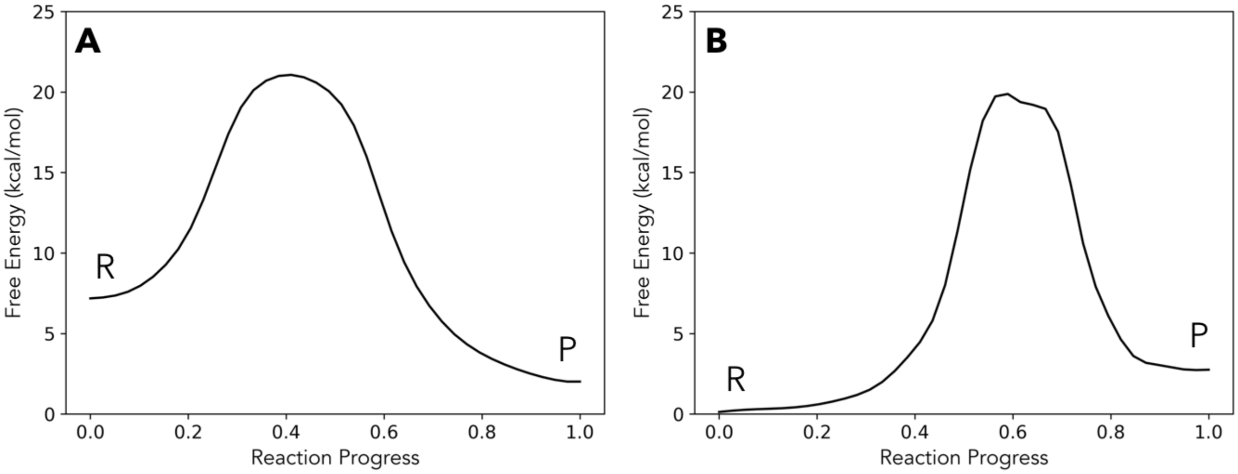

Fig. S6: One-dimensional potentials of mean force (PMF) for acylation and deacylation steps. PMFs along the minimum free energy path (MFEP) for A) acylation reaction and B) deacylation reaction. The MFEPs were computed from the two-dimensional free energy surfaces. These 1D PMFs represent the free energy along the MFEP.

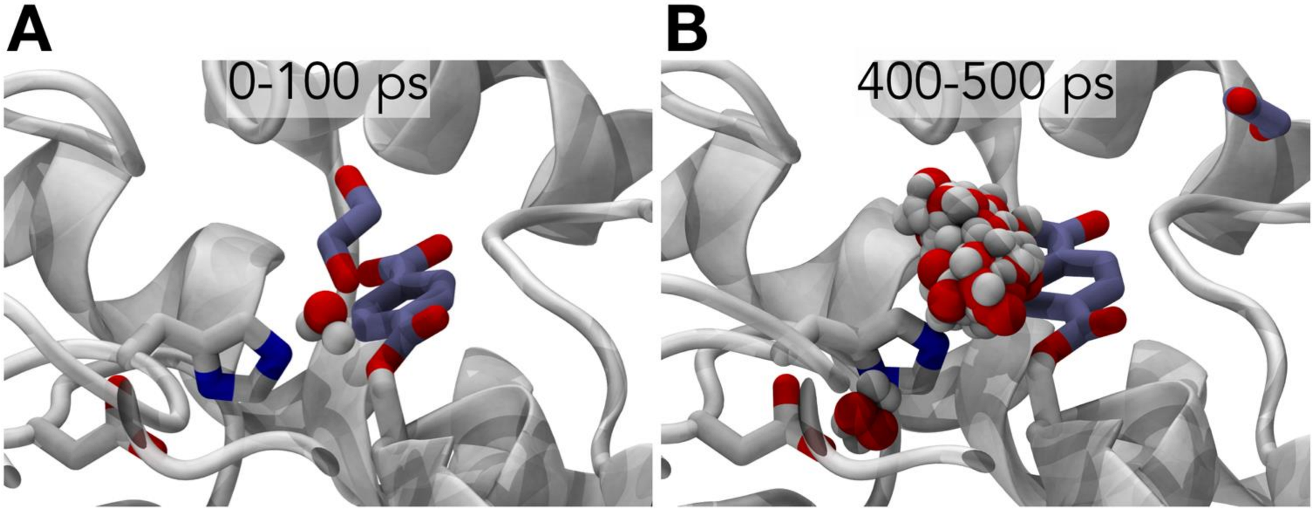

Fig. S7: Post-acylation simulation of active site and reaction products. Water floods the active site after ethylene glycol (EG) leaves the active site post-acylation, as indicated by molecular dynamics simulations. Three independent MD trajectories with classical forcefield were run of the acyl-enzyme intermediate (AEI) immediately following the first chemical step (acylation). In all three simulations, EG leaves the active site within 4 ns. Results from one such trajectory are shown A) in the first 100 ps after acylation and B) in the time period 400-500 ps after acylation. EG leaves the active site in the intervening time. Water molecules within 3 Å of both the carbonyl carbon of the AEI *and* NE2 atom of His528 are shown every 2 ps. The backbone trace and catalytic residues of MHETase are shown in white cartoon and sticks representation, respectively. Purple sticks show the terephthalic acid moiety of the AEI and EG. Analysis and image were created in VMD (57).

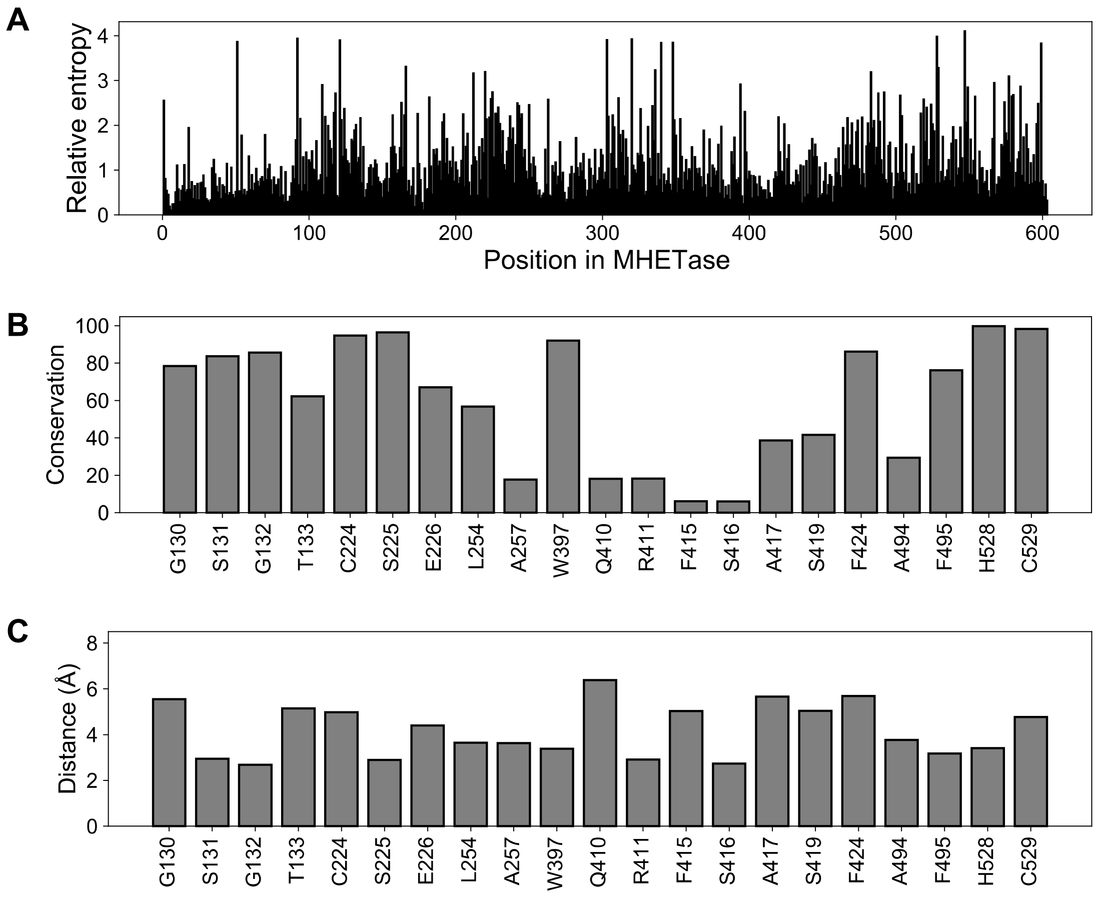

Fig. S8: Conservation analysis of 6,671 tannase family sequences. A) Conservation scores (relative entropy) of positions in tannase family sequences, plotted against the 603 positions in MHETase. A higher relative entropy implies a greater level of amino acid conservation in the site. B) Conservation scores of active-site residues in MHETase within 6 Å of the MHET substrate, including Gln410 (6.3 Å). Conservation scores are shown as percentiles. Ala257, Gln410, Arg411, Phe415, and Ser416 are the least conserved active-site positions in the active site and are more variable than 81% of all positions in MHETase. C) Closest distance between atoms of MHETase active-site residues and the MHET substrate. The molecular coordinates for MHETase bound with MHET are the same as those in the model from which the molecular simulations were started, as described in the Supplementary Methods above.

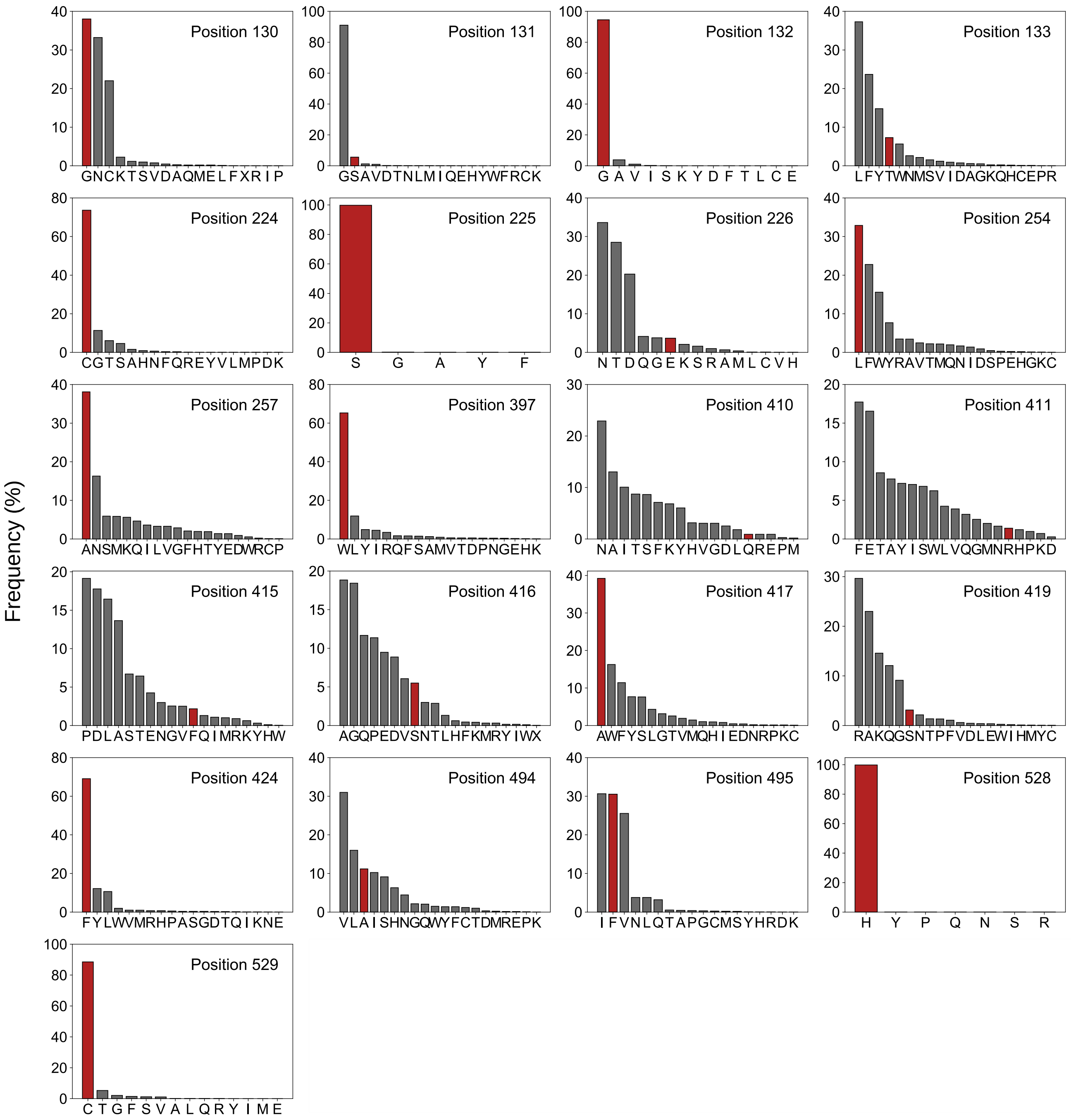

Fig. S9: Amino acid frequencies of active-site positions in MHETase within 6 Å of the MHET substrate, including Gln410 (6.3 Å). The frequency of amino acids for each position was determined from a MAFFT multiple sequence alignment of 6,671 tannase family sequences. The positions are named using *Is* MHETase numbering, and the red bars indicate the amino acids in *Is* MHETase.

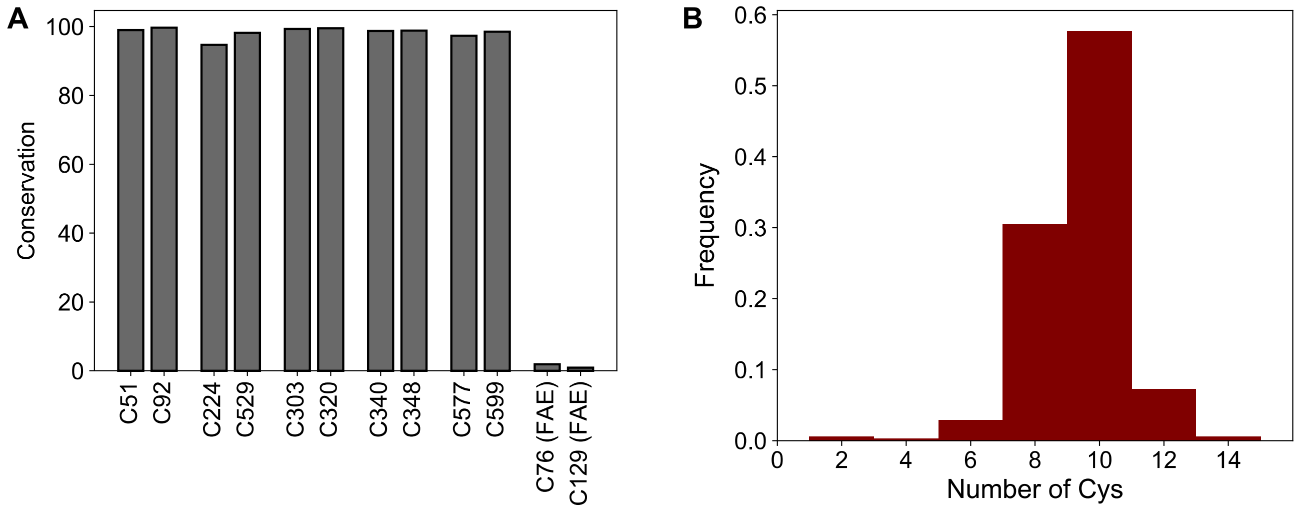

Fig. S10: Disulfide bond cysteines in 6,671 tannase family sequences. A) Conservation of Cys positions forming five disulfide bonds in MHETase. Conservation scores are shown as percentiles. *Ao* FAEB-1 has a 6th disulfide bond between Cys76 and Cys129 which are very variable positions and are less conserved than 98% of positions in multiple sequence alignment. B) Histogram of Cys occurrence in tannase family sequences showing the rarity of a 6th disulfide bond. Assuming, all Cys form disulfide bonds, less than 8% of tannase family sequences have six disulfide bonds.

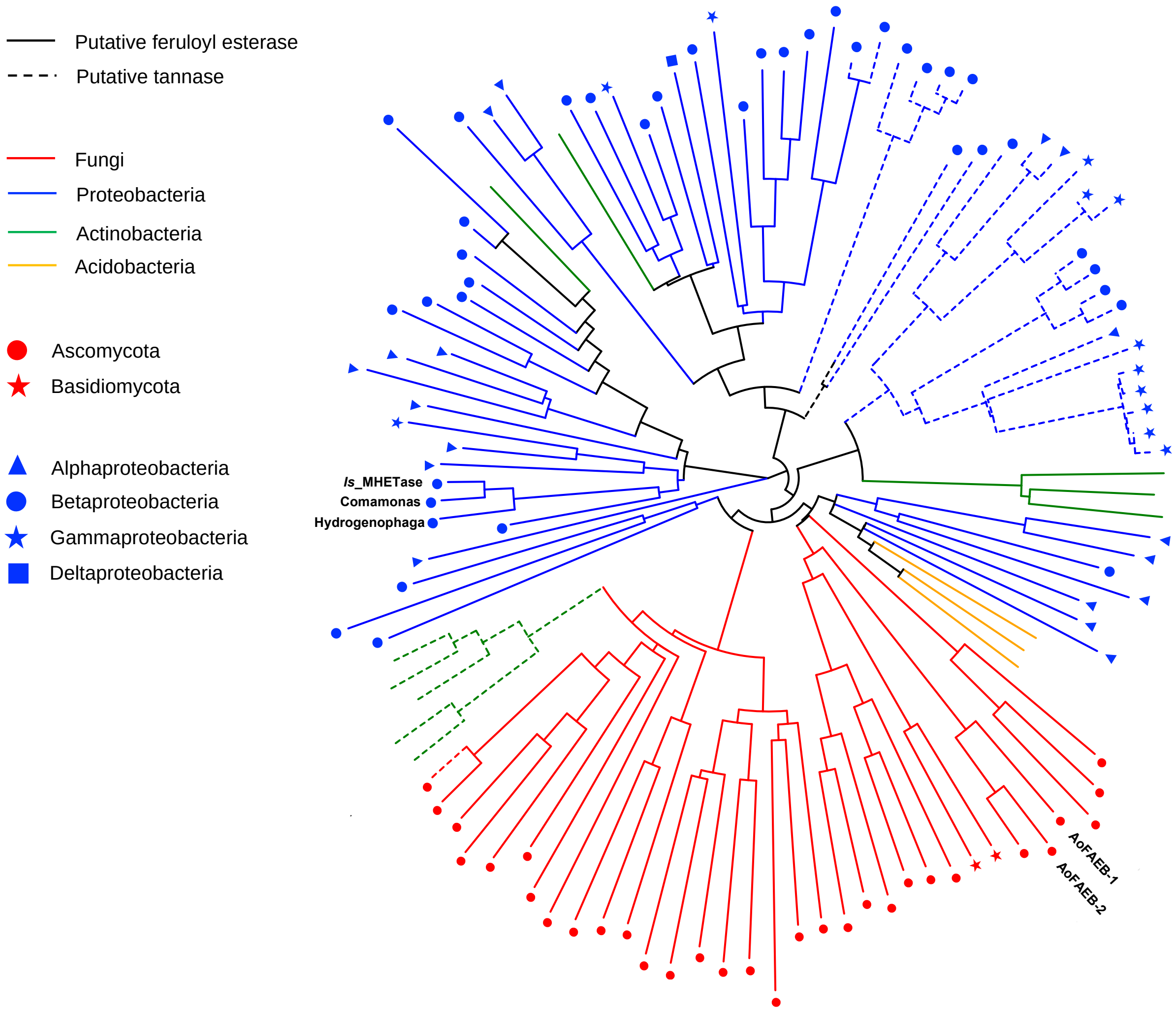

Fig. S11: Phylogenetic analysis of 120 tannase family sequences with minimum evolution method and 1000 bootstrap replicates. Nodes with bootstrap values between 75% and 100% are indicated with gray circles having sizes that are proportional to the bootstrap values. Multiple sequence alignment was conducted with MAFFT, and the phylogenetic analysis was conducted with MEGA7. *Comamonas* and *Hydrogenophaga* are the close MHETase homolog sequences, with accession codes WP_080747404.1 and WP_083293388.1, respectively. *Ao* FAE-B1 and *Ao* FAE-B2 correspond to the *Aspergillus oryzae* ferulic acid esterases, Q2UP89.1 (PDB 3WMT) and Q2UMX6.1 (PDB 6G21), respectively, which in addition to the recently deposited structures for MHETase (PDB 6QZ1, 6QZ2, 6QZ3, and 6QZ4), the structure from Palm et al. (PDB 6QG9) (14), and *Fusarium oxysporum* (PDB 6FAT), are currently the tannase family sequences with solved crystal structures. From the tree, it is clear that FAEs (solid lines) are more phylogenetically similar to MHETase (also shown in solid line) than tannases (dashed lines).

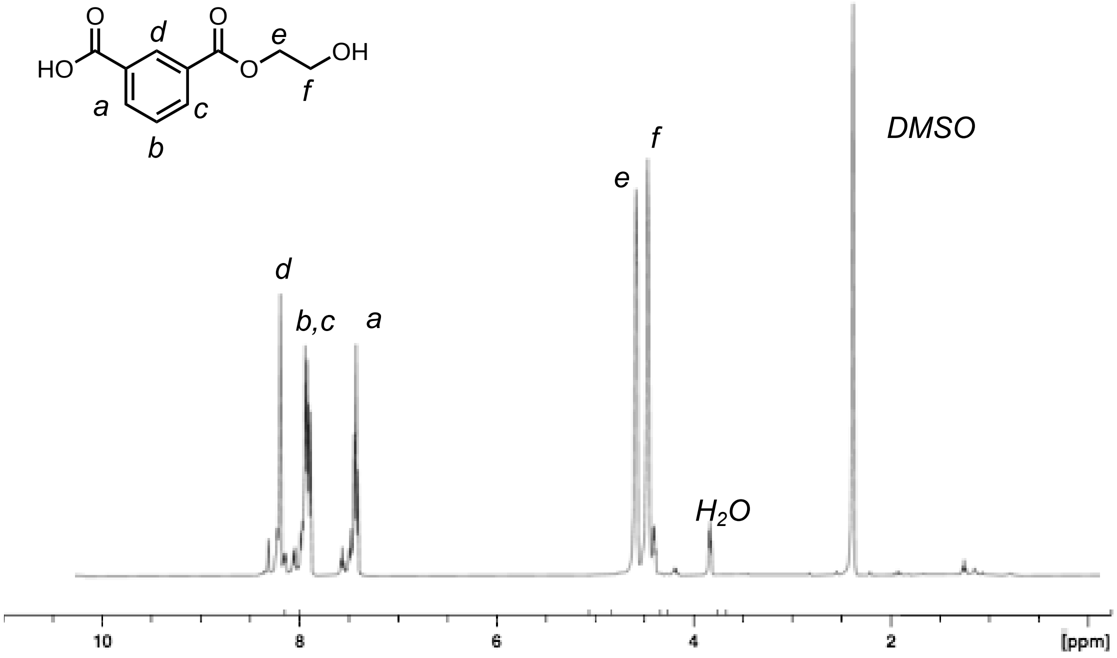

Fig. S12: Validation by NMR of synthesized mono-(2-hydroxytheyl)-isophthalate. ^{1}H NMR spectrum of MHEI with peak assignments. Integration and peak splitting confirm MHEI was formed and that the product is not BHET.

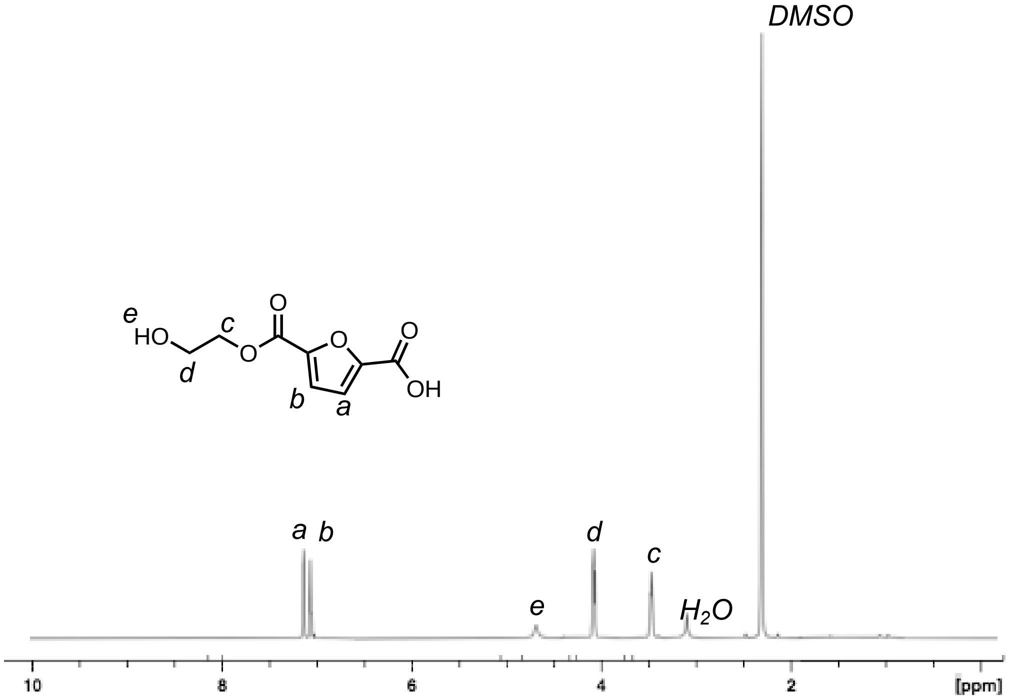

Fig. S13: Validation by NMR of synthesized mono-(2-hydroxytheyl)-furanoate. ^{1}H NMR spectrum of MHEF with peak assignments. Integration and peak splitting confirm MHEF was formed and that the product is not BHET.

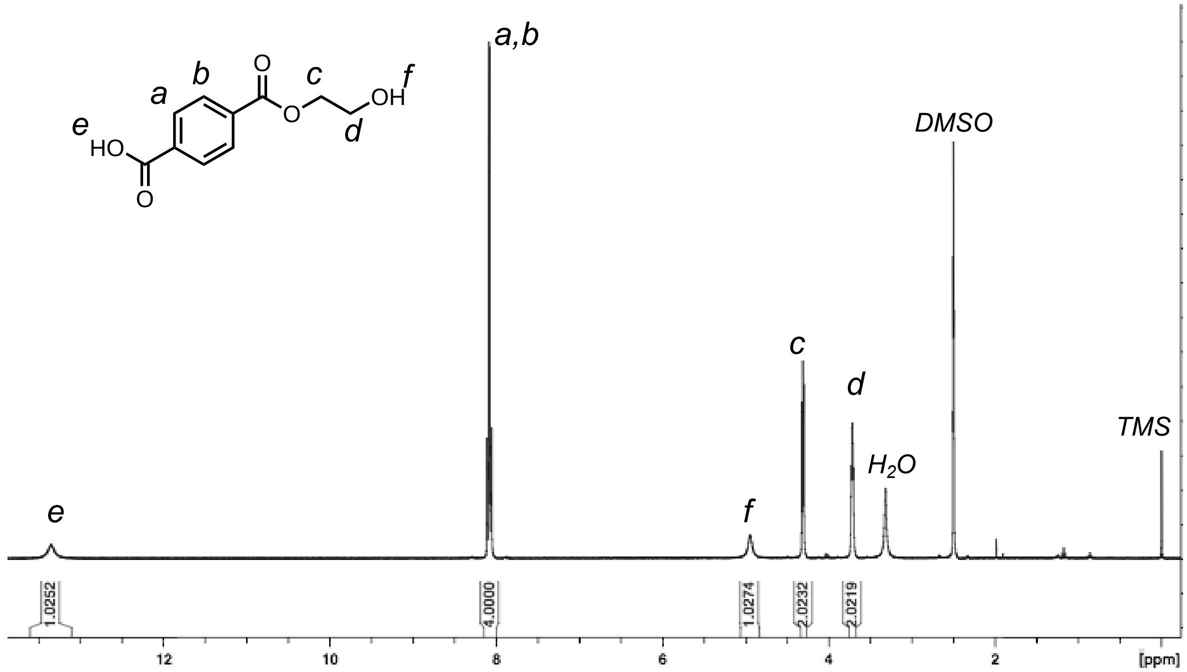

Fig. S14: Validation by NMR of synthesized mono-(2-hydroxytheyl)-terephthalate. ^{1}H NMR spectrum of MHET with peak assignments. Integration and peak splitting confirm MHET was formed and that the product is not BHET.

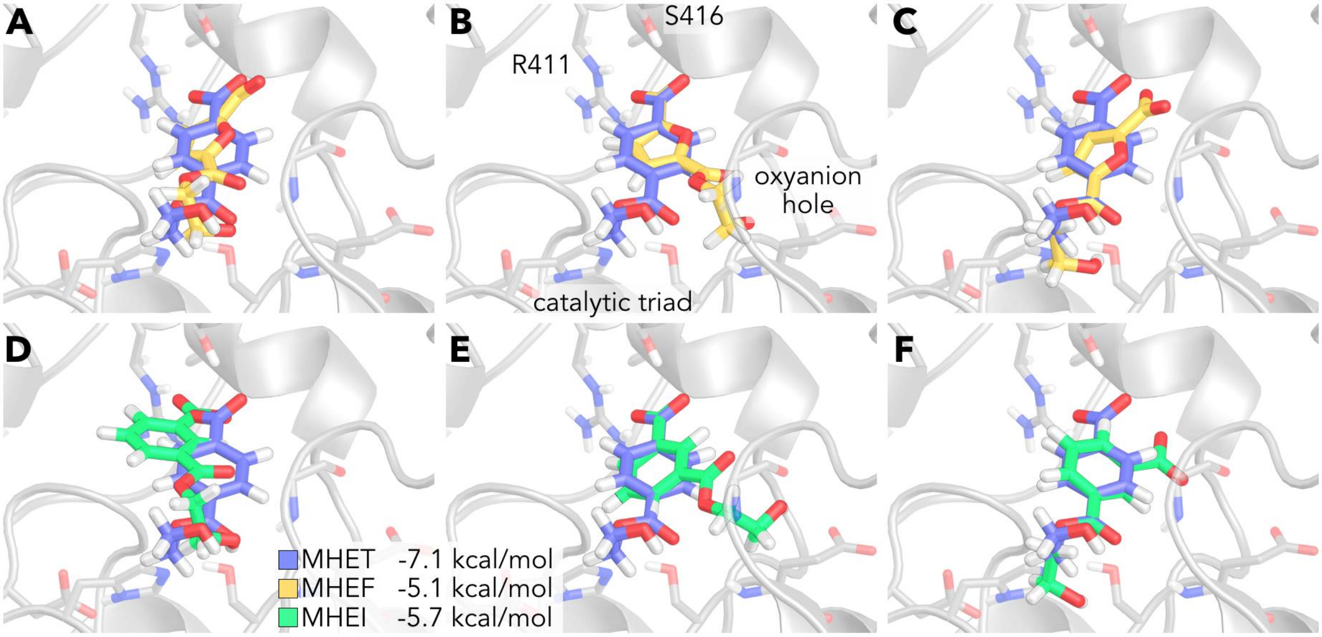

Fig. S15: Flexible molecular docking studies indicate low energy, catalytically active binding mode for MHET, but not MHEF or MHEI. MHET (purple sticks), MHEF (yellow sticks), MHEI (green sticks) from flexible docking studies with MHETase binding site (grey sticks and ribbons) with ligand visualized in three different ways. A) Enzyme backbones aligned bound with MHET and MHEF. B) Alignment of the carboxylate moiety of MHEF to the carboxylate moiety of MHET, in which case the MHEF carbonyl does not lie in the oxyanion hole (as well as the ester bond being located far from the catalytic residues). C) Alignment of the carbonyl of MHEF to the carbonyl of MHET, in which case the carboxylate is out of range to interact with Arg411. D) Enzyme backbones aligned bound with MHET and MHEI. E) Alignment of the carboxylate moiety of MHEI to the carboxylate moiety of MHET, presenting similar issues as with MHEF. F) Alignment of the carbonyl from MHEI to the carbonyl of MHET. The overlaid binding scores represent the lowest energy binding score for catalytically competent poses (i.e. wherein the catalytic triad was intact).

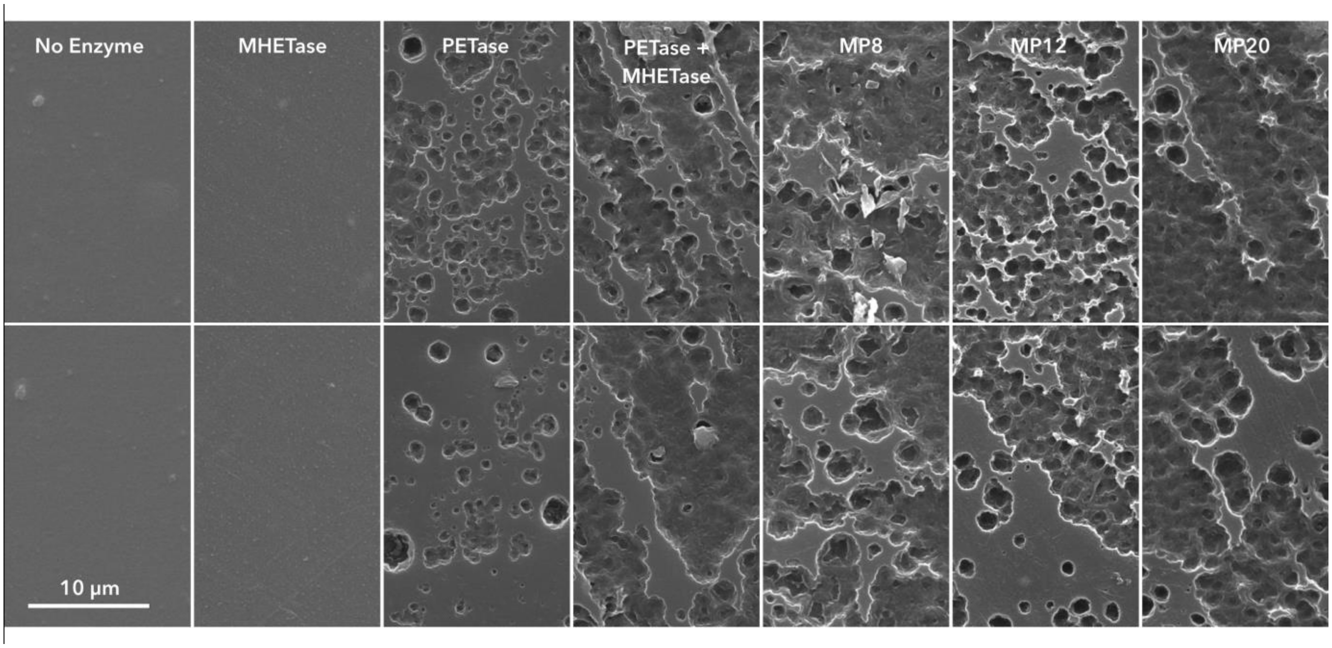

Fig. S16: SEM of amorphous PET film after 96 h enzyme treatment at 30°C. Digestion conditions represent treatment with no enzyme, treatment with 0.4 mg MHETase/g PET, treatment with 0.4 mg PETase/g PET, simultaneous treatment with 0.4 mg PETase and 0.4 mg MHETase/g PET, and treatment with each chimeric enzyme corresponding to samples presented in **Fig. 4D**.

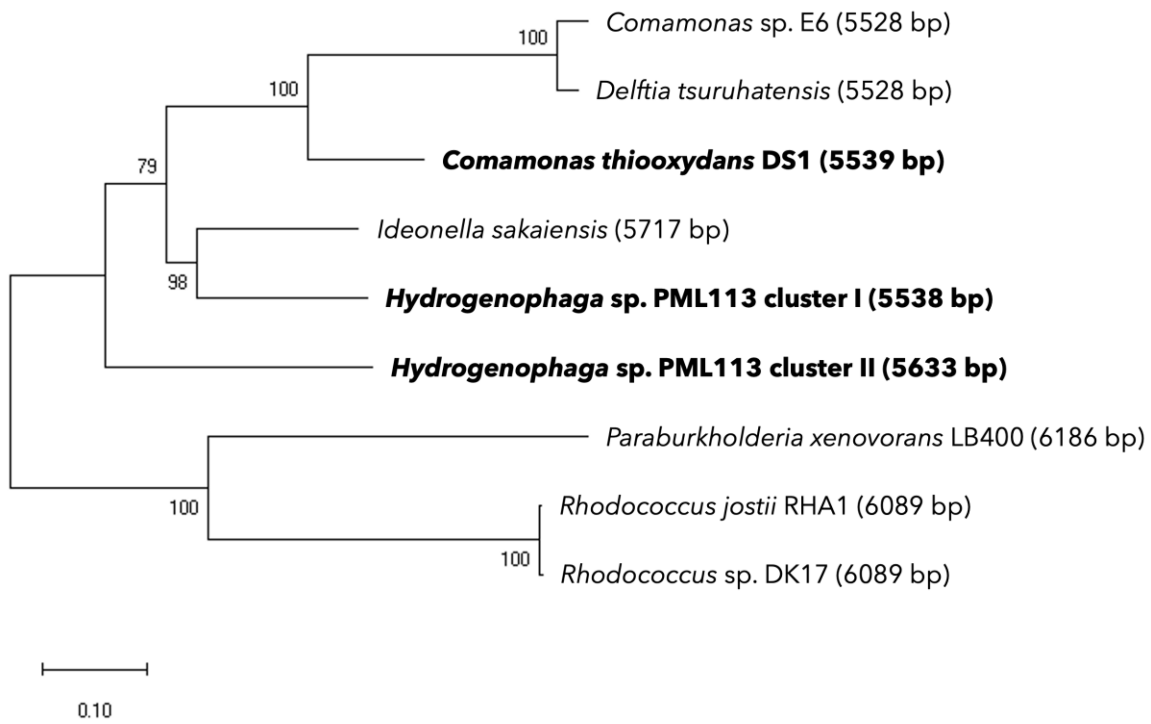

Fig. S17: Evolutionary analysis by Maximum Likelihood method of known and putative TPA gene clusters. MUSCLE multiple sequence alignment of known and putative TPA gene clusters and prediction of the best evolution model were performed using MEGA X (58). The evolutionary history was inferred by using the Maximum Likelihood method and General Time Reversible model (22). The tree with the highest log likelihood (-27899.85) is shown. The percentage of trees in which the associated taxa clustered together is shown next to the branches. Initial tree(s) for the heuristic search were obtained automatically by applying Neighbor-Join and BioNJ algorithms to a matrix of pairwise distances estimated using the Maximum Composite Likelihood (MCL) approach, and then selecting the topology with superior log likelihood value. A discrete Gamma distribution was used to model evolutionary rate differences among sites (5 categories (+*G*, parameter = 0.9655)). The rate variation model allowed for some sites to be evolutionarily invariable ([+*I*], 17.46% sites). The tree is drawn to scale, with branch lengths measured in the number of substitutions per site. This analysis involved 9 nucleotide sequences. All positions with less than 95% site coverage were eliminated, i.e., fewer than 5% alignment gaps, missing data, and ambiguous bases were allowed at any position (partial deletion option). There were a total of 4369 positions in the final dataset. Evolutionary analyses were also conducted in MEGA X. Accession numbers for source sequences used are AB238679 for *Comamonas* sp. E6, FOKN01000001 for *Delftia tsuruhatensis*, NZ_AWTM01000090 for *Comamonas thiooxydans* DS1, NZ_BBYR01000104 for *Ideonella sakaiensis*, NZ_MIYM01000023 and NZ_MIYM01000001 for *Hydrogenophaga* sp. PML113 clusters I and II, respectively, CP000271 for *Paraburkholderia xenovorans* LB400, NC_008269 for *Rhodococcus jostii* RHA1, and AY502076 for *Rhodococcus* sp. DK17.

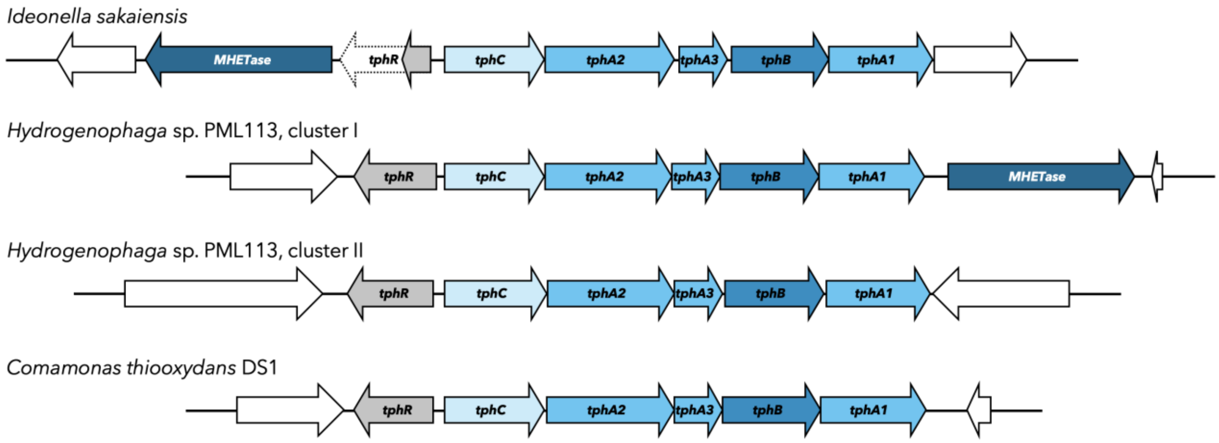

Fig. S18: Schematic representation of putative TPA catabolic gene clusters in *Hydrogenophaga* sp. PML113 and *Comamonas thiooxydans* DS1, compared to *Ideonella sakaiensis*. A frame-shift in the *I. sakaiensis tphR* coding sequence results in a truncated protein. Searches against the genomes of *C. thiooxydans* strains DF1 and DF2 returned partial sequences due to short contig lengths.

**TphA1**		1	2	3	4	5	6	7	8	9
**1**	*Comamonas* sp. E6 TphA1$_{II}$ (BAE47088.1)	100	96	70	59	61	57	44	42	43
**2**	*Delftia tsuruhatensis* TphA1 (WP_034369195.1)		100	70	58	62	57	43	43	44
**3**	*Comamonas thiooxydans* DS1 TphA1 (WP_034398469.1)			100	59	62	60	43	42	42
**4**	*Ideonella sakaiensis* TphA1 (WP_054022750.1)				100	67	60	44	44	44
**5**	*Hydrogenophaga* sp. PML113 TphA1$_{I}$ (WP_070401319.1)					100	58	44	43	43
**6**	*Hydrogenophaga* sp. PML113 TphA1$_{II}$ (WP_070397706.1)						100	48	45	46
**7**	*Paraburkholderia xenovorans* LB400 TphA1 (ABE33246.1)							100	58	58
**8**	*Rhodococcus jostii* RHA1 TphA1 (WP_011599112.1)								100	98
**9**	*Rhodococcus* sp. DK17 TphA1 (AAR90190.1)									100

100% 90% 80% 70% 60% 50% 40%

**TphA2**		1	2	3	4	5	6	7	8	9
**1**	*Comamonas* sp. E6 TphA2$_{II}$ (BAE47085.1)	100	99	88	78	81	75	63	68	67
**2**	*Delftia tsuruhatensis* TphA2 (WP_047219860.1)		100	88	79	81	77	64	68	67
**3**	*Comamonas thiooxydans* DS1 TphA2 (WP_034398463.1)			100	79	83	77	64	67	67
**4**	*Ideonella sakaiensis* TphA2 (WP_054022747.1)				100	82	81	66	69	69
**5**	*Hydrogenophaga* sp. PML113 TphA2$_{I}$ (WP_070401317.1)					100	80	65	68	68
**6**	*Hydrogenophaga* sp. PML113 TphA2$_{II}$ (WP_083292878.1)						100	63	66	66
**7**	*Paraburkholderia xenovorans* LB400 TphA2 (ABE33243.1)							100	71	71
**8**	*Rhodococcus jostii* RHA1 TphA2 (WP_011599110.1)								100	100
**9**	*Rhodococcus* sp. DK17 TphA2 (AAR90187.1)									100

**TphA3**		1	2	3	4	5	6	7	8	9
**1**	*Comamonas* sp. E6 TphA3$_{II}$ (BAE47086.1)	100	99	80	67	68	64	49	49	49
**2**	*Delftia tsuruhatensis* TphA3 (WP_034369201.1)		100	81	68	69	65	49	49	49
**3**	*Comamonas thiooxydans* DS1 TphA3 (WP_034398465.1)			100	73	74	71	49	53	53
**4**	*Ideonella sakaiensis* TphA3 (WP_054022748.1)				100	78	75	52	52	52
**5**	*Hydrogenophaga* sp. PML113 TphA3$_{I}$ (WP_070401318.1)					100	77	47	53	53
**6**	*Hydrogenophaga* sp. PML113 TphA3$_{II}$ (WP_070397704.1)						100	48	52	52
**7**	*Paraburkholderia xenovorans* LB400 TphA3 (BE33244.1)							100	56	56
**8**	*Rhodococcus jostii* RHA1 TphA3 (WP_005256013.1)								100	100
**9**	*Rhodococcus* sp. DK17 TphA3 (AAR90188.1)									100

**TphB**		1	2	3	4	5	6	7	8	9
**1**	*Comamonas* sp. E6 TphB$_{II}$ (BAE47087.1)	100	96	77	65	67	58	48	49	48
**2**	*Delftia tsuruhatensis* TphB (WP_047219859.1)		100	76	65	66	58	47	48	48
**3**	*Comamonas thiooxydans* DS1 TphB (WP_034398467.1)			100	68	70	62	49	48	48
**4**	*Ideonella sakaiensis* TphB (WP_082368716.1)				100	74	67	53	51	49
**5**	*Hydrogenophaga* sp. PML113 TphB 23 (WP_070401322.1)					100	62	50	46	45
**6**	*Hydrogenophaga* sp. PML113 TphB 1 (WP_070397705.1)						100	51	48	47
**7**	*Paraburkholderia xenovorans* LB400 TphB (ABE33245.1)							100	51	50
**8**	*Rhodococcus jostii* RHA1 TphB (WP_011599111.1)								100	99
**9**	*Rhodococcus* sp. DK17 TphB (AAR90189.1)									100

Fig. S19: Sequence identity matrices for putative TPA catabolic proteins. Identity values obtained from pairwise alignments performed by Clustal Omega (59).

Supplementary Tables

Table S1. Crystallographic data, model refinement, and crystallization conditions of *Is*-MHETase.

Data set	SeMet	Native 1	Native 2	Native 3
Space Group	$P22_12_1$	$P22_12_1$	$P2_12_12_1$	$P1$
Wavelength (Å)	0.9795	0.9795	0.9795	0.9795
Resolution Range (Å)	49.30 - 1.60	46.00 - 1.70	46.24 - 1.80	95.29 - 1.90
Unique reflections	84000	70855	122377	506864
Completeness (%)[a]	99.8 (98.9)	99.6 (98.0)	98.2 (96.9)	93.7 (92.4)
Anomalous Completeness (%)[a]	99.8 (98.5)			
*R*merge[b]	0.063 (0.511)	0.056 (0.584)	0.067 (0.550)	0.057 (0.254)
CC(1/2)[c]	0.999 (0.902)	0.999 (0.900)	0.999 (0.914)	
Multiplicity[d]	12.6 (11.0)	6.4 (6.5)	6.7 (6.6)	1.7 (1.7)
Anomalous Multiplicity[d]	6.6 (5.6)			
I/σI[a]	20.2 (4.6)	15.4 (3.1)	14.7 (2.9)	7.1 (2.6)
	a = 77.37 Å, b = 89.02 Å, c = 91.64 Å	a = 77.20 Å, b = 89.88 Å, c = 92.00 Å	a = 90.21 Å, b = 92.80 Å, c = 159.99 Å	a = 110.49 Å, b = 135.63 Å, c = 138.15 Å, α = 83.09°, β = 67.91°, γ = 67.57°
Model Refinement				
Resolution Range (Å)	45.82 - 1.60	46.00 – 1.70	46.24 - 1.80	46.46 – 1.90
No. of residues:	A: 40-55, 62-603	A: 40-55, 61-603	A: 36-603, B: 36-603	A: 42-603 , B:43-603, C: 43-603, D: 43-603, E: 43-603, F: 43-603, G: 41-603, H: 42-603, I: 43-603, J: 43-603
No. of water, ligands	748, 1 Ca, 1 benzoic acid	552, 1 Ca, 1 benzoic acid	1407, 2 Ca	6125, 10 Ca
Rwork/Rfree (%)[e]	16.20 (17.80)	16.45 (19.18)	18.24 (20.51)	18.54 (20.54)
B average[f]	25.8	32.5	30.9	28.8
Geometry bond, angles[g]	0.003, 0.613	0.008, 0.900	0.005, 0.715	0.003, 0.576
Ramachandran[h]	97.47, 0.0	97.48, 0.2	97.0, 0.0	97.07, 0.02
Molprobity Clash Score	1.61	1.85	3.70	4.15
Beamline	I03	I03	I03	I03
PDB ID[i]	6QZ3	6QZ1	6QZ4	6QZ2

[a] Signal to noise ratio of intensities, highest resolution bin in brackets. [b] R_m : $\sum h \sum i |I/(h,i) - I(h)| / \sum h \sum i / I(h,i)$ where $I(h,i)$ are symmetry-related intensities and $I(h)$ is the mean intensity of the reflection with unique index h . [c] $CC_{1/2}$ is the correlation coefficient of the mean intensities between two random half-datasets. [d] Multiplicity for unique reflections. [e] 5% of reflections were randomly selected for determination of the free R factor, prior to any refinement. [f] Temperature factors averaged for all atoms. [g] RMS deviations from ideal geometry for bond lengths and restraint angles (9). [h] Percentage of residues in the 'most favoured region' of the Ramachandran plot and percentage of outliers (MOLPROBITY) (60). [i]Protein Data Bank identifiers for coordinates.

Crystallography conditions: SeMet; 0.1 M sodium cacodylate (pH 6.5), 9% PEG 8000. Native 1; 0.1 M sodium acetate (pH 5.5), 24% PEG 5000 MME. Native 2; ammonium acetate (pH 4.5), 22.5% PEG 10000. Native 3; 0.1 M sodium citrate (pH 5.5), 1.0 M ammonium phosphate monobasic. All conditions used 20% glycerol as a cryoprotectant.

Table S2. Tannase family sequences used in phylogenetic analysis.

	Accession	Annotation	Organism	Taxon
1	A0A0K8P8E7.1	mono(2-hydroxyethyl) terephthalate hydrolase	*Ideonella sakaiensis*	Betaproteobacteria
2	WP_080747404.1	tannase/feruloyl esterase family alpha/beta	*Comamonas thiooxydans*	Betaproteobacteria
3	WP_083293388.1	tannase/feruloyl esterase family alpha/beta	*Hydrogenophaga sp.*	Betaproteobacteria
4	Q2UP89.1	probable feruloyl esterase b-1	*Aspergillus oryzae*	Ascomycota
5	Q2UMX6.1	Probable feruloyl esterase B-2	*Aspergillus oryzae*	Ascomycota
6	KQO20166.1	feruloyl esterase	*Acidovorax sp.*	Betaproteobacteria
7	SFM74645.1	feruloyl esterase	*Bradyrhizobium sp.*	Alphaproteobacteria
8	EGC99108.1	feruloyl esterase	*Burkholderia sp.*	Betaproteobacteria
9	RLJ38044.1	feruloyl esterase	*Acidovorax sp.*	Betaproteobacteria
10	RKR69440.1	feruloyl esterase	*Acidovorax sp.*	Betaproteobacteria
11	SOD27033.1	feruloyl esterase	*Variovorax sp.*	Betaproteobacteria
12	REF22346.1	feruloyl esterase	*Microbacterium trichothecenolyticum*	Actinobacteria
13	RAR84815.1	feruloyl esterase	*Acidovorax anthurii*	Betaproteobacteria
14	ALV26718.1	feruloyl esterase	*Pannonibacter phragmitetus*	Alphaproteobacteria
15	ODT65815.1	feruloyl esterase	*Pelagibacterium sp.*	Alphaproteobacteria
16	KMO18581.1	feruloyl esterase	*Methylobacterium platani*	Alphaproteobacteria
17	SYX90233.1	putative feruloyl esterase b-1	*Pseudomonas reidholzensis*	Gammaproteobacteria
18	SFO05094.1	feruloyl esterase	*Formivibrio citricus*	Betaproteobacteria
19	SFV19457.1	feruloyl esterase	*Bradyrhizobium arachidis*	Alphaproteobacteria
20	OYX09584.1	feruloyl esterase	*Rhizobiales bacterium*	Alphaproteobacteria
21	OLB33458.1	feruloyl esterase	*Acidobacteria bacterium*	unclassified Acidobacteria
22	OLD21188.1	feruloyl esterase	*Acidobacteria bacterium*	unclassified Acidobacteria
23	SEB13840.1	feruloyl esterase	*Variovorax sp.*	Betaproteobacteria
24	SDU16980.1	feruloyl esterase	*Amycolatopsis keratiniphila*	Actinobacteria
25	SEM06276.1	feruloyl esterase	*Variovorax sp.*	Betaproteobacteria
26	RKT74992.1	feruloyl esterase	*Saccharothrix variisporea*	Actinobacteria
27	PJJ32606.1	feruloyl esterase	*Afipia broomeae*	Alphaproteobacteria
28	CCE09743.1	putative feruloyl esterase	*Bradyrhizobium sp.*	Alphaproteobacteria
29	KVV28346.1	feruloyl esterase	*Burkholderia multivorans*	Betaproteobacteria
30	KXU82530.1	feruloyl esterase	*Paraburkholderia monticola*	Betaproteobacteria
31	OYX06871.1	feruloyl esterase	*Sphingomonadales bacterium*	Alphaproteobacteria
32	ACC69322.1	feruloyl esterase	*Paraburkholderia phymatum*	Betaproteobacteria
33	PVX62318.1	feruloyl esterase	*Sphingomonas sp.*	Alphaproteobacteria
34	ETF04378.1	feruloyl esterase	*Advenella kashmirensis*	Betaproteobacteria
35	KVR34266.1	feruloyl esterase	*Burkholderia ubonensis*	Betaproteobacteria
36	SOB89932.1	feruloyl esterase	*Alcanivorax xenomutans*	Gammaproteobacteria
37	KPV20554.1	feruloyl esterase	*Variovorax paradoxus*	Betaproteobacteria
38	OFW45315.1	feruloyl esterase	*Acidobacteria bacterium*	unclassified Acidobacteria
39	RKD63451.1	feruloyl esterase	*Caballeronia udeis*	Betaproteobacteria
40	SEO24475.1	feruloyl esterase	*Bradyrhizobium sp.*	Alphaproteobacteria
41	ODU08862.1	feruloyl esterase	*Rubrivivax sp.*	Betaproteobacteria
42	KOV79372.1	feruloyl esterase	*Nocardia sp.*	Actinobacteria
43	OYZ97564.1	feruloyl esterase	*Novosphingobium sp.*	Alphaproteobacteria
44	ORY14570.1	feruloyl esterase b precursor	*Clohesyomyces aquaticus*	Ascomycota

45	SCK19622.1	feruloyl esterase	*Variovorax sp.*	Betaproteobacteria
46	PYG17545.1	feruloyl esterase	*Novosphingobium sp.*	Alphaproteobacteria
47	SAK87127.1	feruloyl esterase	*Caballeronia fortuita*	Betaproteobacteria
48	RAR95830.1	feruloyl esterase	*Rahnella sp.*	Gammaproteobacteria
49	AQQ72604.1	feruloyl esterase	*Talaromyces piceae*	Ascomycota
50	SEI21695.1	feruloyl esterase	*Paraburkholderia hospita*	Betaproteobacteria
51	XP_002341414.1	feruloyl esterase, putative	*Talaromyces stipitatus*	Ascomycota
52	PZQ62240.1	feruloyl esterase	*Variovorax paradoxus*	Betaproteobacteria
53	XP_016588953.1	feruloyl esterase	*Sporothrix schenckii*	Ascomycota
54	ODU17436.1	feruloyl esterase	*Variovorax sp.*	Betaproteobacteria
55	ENH84593.1	feruloyl esterase b	*Colletotrichum orbiculare*	Ascomycota
56	SDF78426.1	feruloyl esterase	*Lechevalieria fradiae*	Actinobacteria
57	ESZ94426.1	feruloyl esterase b precursor	*Sclerotinia borealis*	Ascomycota
58	CUA69813.1	putative feruloyl esterase b-2	*Rhizoctonia solani*	Basidiomycota
59	ORY10730.1	feruloyl esterase b precursor	*Clohesyomyces aquaticus*	Ascomycota
60	PIG83702.1	feruloyl esterase b precursor	*Aspergillus arachidicola*	Ascomycota
61	KYF55538.1	feruloyl esterase	*Sorangium cellulosum*	delta/epsilon subdivisions
62	XP_002844880.1	feruloyl esterase b	*Microsporum canis*	Ascomycota
63	KFG78502.1	putative feruloyl esterase	*Metarhizium anisopliae*	Ascomycota
64	SDC72403.1	feruloyl esterase	*Cupriavidus sp.*	Betaproteobacteria
65	XP_018178631.1	feruloyl esterase b	*Purpureocillium lilacinum*	Ascomycota
66	XP_009650693.1	feruloyl esterase b	*Verticillium dahliae*	Ascomycota
67	CEL56090.1	putative feruloyl esterase b-1 os=aspergillus	*Rhizoctonia solani*	Basidiomycota
68	OJW26926.1	feruloyl esterase	*Sphingopyxis sp.*	Alphaproteobacteria
69	XP_013423336.1	putative feruloyl esterase	*Aureobasidium namibiae*	Ascomycota
70	SFT75211.1	feruloyl esterase	*Paraburkholderia aspalathi*	Betaproteobacteria
71	XP_025497975.1	feruloyl esterase b precursor	*Aspergillus aculeatinus*	Ascomycota
72	GAT22098.1	feruloyl esterase b precursor	*Aspergillus luchuensis*	Ascomycota
73	SFG45211.1	feruloyl esterase	*Novosphingobium sp.*	Alphaproteobacteria
74	SIN83151.1	feruloyl esterase	*Paraburkholderia phenazinium*	Betaproteobacteria
75	KNG46319.1	feruloyl esterase b precursor	*Stemphylium lycopersici*	Ascomycota
76	OAQ87654.1	feruloyl esterase	*Purpureocillium lilacinum*	Ascomycota
77	PMD29264.1	putative ferulic acid esterase	*Hyaloscypha variabilis*	Ascomycota
78	GCB21914.1	probable feruloyl esterase arb_07085	*Aspergillus awamori*	Ascomycota
79	XP_001932100.1	feruloyl esterase b precursor	*Pyrenophora tritici-repentis*	Ascomycota
80	EMT66649.1	putative feruloyl esterase b-2	*Fusarium oxysporum*	Ascomycota
81	XP_025508882.1	feruloyl esterase	*Aspergillus aculeatinus*	Ascomycota
82	OAQ76531.1	feruloyl esterase	*Purpureocillium lilacinum*	Ascomycota
83	KLU81119.1	feruloyl esterase b	*Magnaporthiopsis poae*	Ascomycota
84	OMP83047.1	feruloyl esterase b	*Diplodia seriata*	Ascomycota
85	PTD08579.1	putative feruloyl esterase	*Fusarium culmorum*	Ascomycota
86	RKT10778.1	feruloyl esterase	*Paraburkholderia sp.*	Betaproteobacteria
87	XP_025430110.1	putative feruloyl esterase b-2	*Aspergillus saccharolyticus*	Ascomycota
88	XP_025492021.1	feruloyl esterase	*Aspergillus uvarum*	Ascomycota
89	ODU15911.1	feruloyl esterase	*Variovorax sp.*	Betaproteobacteria
90	OXC77937.1	tannase precursor	*Caballeronia sordidicola*	Betaproteobacteria
91	KPX81997.1	tannase	*Pseudomonas meliae*	Gammaproteobacteria

92	RMP50230.1	tannase	*Pseudomonas savastanoi*	Gammaproteobacteria
93	RMV15745.1	tannase	*Pseudomonas savastanoi*	Gammaproteobacteria
94	KPX21055.1	tannase	*Pseudomonas amygdali*	Gammaproteobacteria
95	SPD56196.1	tannase	*Cupriavidus taiwanensis*	Betaproteobacteria
96	BAQ47614.1	tannase	*Methylobacterium aquaticum*	Alphaproteobacteria
97	OTP71705.1	tannase precursor	*Caballeronia sordidicola*	Betaproteobacteria
98	AEA83596.1	tannase precursor	*Pseudomonas stutzeri*	Gammaproteobacteria
99	OUI87529.1	tannase	*Acetobacter sp.*	Alphaproteobacteria
100	KQP45511.1	tannase	*Pseudorhodoferax sp.*	Betaproteobacteria
101	AUB50195.1	tannase precursor	*Klebsiella pneumoniae*	Gammaproteobacteria
102	OAJ68072.1	tannase	*Gluconobacter cerinus*	Alphaproteobacteria
103	ERK18637.1	tannase precursor	*Pantoea sp.*	Gammaproteobacteria
104	CDL19993.1	tannase precursor	*Klebsiella pneumoniae*	Gammaproteobacteria
105	OAG73259.1	tannase	*Gluconobacter japonicus*	Alphaproteobacteria
106	KRC31825.1	tannase	*Acidovorax sp.*	Betaproteobacteria
107	BAU88379.1	tannase	*Streptomyces laurentii*	Actinobacteria
108	KMS92636.1	tannase	*Streptomyces regensis*	Actinobacteria
109	KAK47681.1	tannase	*Caballeronia jiangsuensis*	Betaproteobacteria
110	AKN72753.1	tannase	*Streptomyces sp.*	Actinobacteria
111	OLL31673.1	tannase	*Burkholderia sp.*	Betaproteobacteria
112	KDR33867.1	tannase	*Caballeronia zhejiangensis*	Betaproteobacteria
113	KQB59229.1	tannase	*Acidovorax sp.*	Betaproteobacteria
114	XP_003014523.1	tannase, putative	*Trichophyton benhamiae*	Ascomycota
115	KZT17459.1	tannase	*Acidovorax sp.*	Betaproteobacteria
116	KQO35844.1	tannase	*Acidovorax sp.*	Betaproteobacteria
117	KXU83547.1	tannase	*Paraburkholderia monticola*	Betaproteobacteria
118	KOV87180.1	tannase	*Nocardia sp.*	Actinobacteria
119	KJK45095.1	tannase	*Lechevalieria aerocolonigenes*	Actinobacteria
120	KEZ68139.1	tannase	*Pseudomonas amygdali*	Gammaproteobacteria

Table S3. Michaelis-Menten kinetic parameters

Enzyme	K_m	V_{max}	K_i	R^2	k_{cat}/ K_m
	(μM)	(μM s^{-1})	(μM)		(μM^{-1} s^{-1})
Is MHETase	23.17±1.65	0.25±0.05	307.30±20.65	0.90	2.17
Is MHETase S131G	184.10±3.50	0.11±0.03	-	0.93	0.06
Comamonas thiooxydans	174.70±4.75	0.20±0.05	78.80±3.04	0.93	0.23
Hydrogenophaga sp. PML113	41.09±3.38	0.01±0.00	221.50±19.01	0.93	0.13

Results of fitting initial reaction velocities of enzymatic turnover of substrate concentrations between 10 μM and 250 μM using Michaelis-Menten models. The model with substrate inhibition is used for *Is* MHETase, *Comamonas thiooxydans*, and *Hydrogenophage* sp. PML113, while the classic Michaelis-Menten model is used for *Is* MHETase S131G. Non-linear regression performed using GraphPad Prism (8.4.1) is reported, along with 95% confidence intervals for each parameter and R^2 value given for fit of the model to the data.

Table S4. Synergistic degradation of amorphous PET film.

PETase Loading	MHETase Loading	TPA		MHET		BHET		Sum Products	
		Average	St. Dev.	Average	St. Dev.	Average	St. Dev.	Sum	St. Dev.
(mg Enzyme /g PET)		(mM)		(mM)		(mM)		(mM)	
0	0	0.00	0.00	0.00	0.00	0.00	0.00	0.00	0.00
0	0.1	0.00	0.00	0.00	0.00	0.00	0.00	0.00	0.00
0	0.2	0.00	0.00	0.00	0.00	0.00	0.00	0.00	0.00
0	0.3	0.00	0.00	0.00	0.00	0.00	0.00	0.00	0.00
0	0.4	0.00	0.00	0.00	0.00	0.00	0.00	0.00	0.00
0	0.5	0.00	0.00	0.00	0.00	0.00	0.00	0.00	0.00
0	0.6	0.00	0.00	0.00	0.00	0.00	0.00	0.00	0.00
0	0.8	0.00	0.00	0.00	0.00	0.00	0.00	0.00	0.00
0	1	0.00	0.00	0.00	0.00	0.00	0.00	0.00	0.00
0.1	0	0.01	0.00	0.02	0.01	0.00	0.00	0.02	0.01
0.1	0.1	0.11	0.03	0.00	0.00	0.00	0.00	0.12	0.03
0.1	0.2	0.19	0.02	0.00	0.00	0.00	0.00	0.19	0.02
0.1	0.3	0.27	0.03	0.00	0.00	0.00	0.00	0.27	0.03
0.1	0.4	0.30	0.05	0.00	0.00	0.00	0.00	0.30	0.05
0.1	0.5	0.27	0.05	0.00	0.00	0.00	0.00	0.28	0.05
0.1	0.6	0.22	0.03	0.00	0.00	0.00	0.00	0.23	0.03
0.1	0.8	0.25	0.02	0.00	0.00	0.00	0.00	0.25	0.02
0.1	1	0.27	0.08	0.00	0.00	0.00	0.00	0.28	0.08
0.2	0	0.03	0.01	0.06	0.01	0.00	0.00	0.10	0.02
0.2	0.1	0.44	0.05	0.00	0.00	0.00	0.00	0.44	0.05
0.2	0.2	0.41	0.12	0.00	0.00	0.00	0.00	0.42	0.12
0.2	0.3	0.44	0.10	0.00	0.00	0.00	0.00	0.45	0.10
0.2	0.4	0.49	0.04	0.00	0.00	0.00	0.00	0.49	0.04
0.2	0.5	0.53	0.06	0.00	0.00	0.00	0.00	0.53	0.06
0.2	0.6	0.40	0.08	0.00	0.00	0.00	0.00	0.40	0.08
0.2	0.8	0.29	0.01	0.00	0.00	0.00	0.00	0.30	0.01
0.2	1	0.37	0.17	0.00	0.00	0.00	0.00	0.37	0.17
0.3	0	0.09	0.00	0.15	0.00	0.00	0.00	0.23	0.01
0.3	0.1	0.61	0.07	0.00	0.00	0.00	0.00	0.61	0.07
0.3	0.2	0.76	0.02	0.00	0.00	0.00	0.00	0.76	0.02
0.3	0.3	0.85	0.08	0.00	0.00	0.00	0.00	0.85	0.08
0.3	0.4	0.80	0.15	0.00	0.00	0.00	0.00	0.81	0.15
0.3	0.5	0.89	0.03	0.00	0.00	0.00	0.00	0.89	0.03
0.3	0.6	0.86	0.13	0.00	0.00	0.00	0.00	0.86	0.13
0.3	0.8	0.89	0.07	0.00	0.00	0.00	0.00	0.89	0.07
0.3	1	0.81	0.12	0.00	0.00	0.00	0.00	0.81	0.12
0.4	0	0.14	0.01	0.19	0.01	0.00	0.00	0.33	0.02
0.4	0.1	0.93	0.15	0.00	0.00	0.00	0.00	0.93	0.15
0.4	0.2	1.03	0.15	0.00	0.00	0.00	0.00	1.03	0.15
0.4	0.3	1.06	0.06	0.00	0.00	0.00	0.00	1.06	0.06
0.4	0.4	1.07	0.04	0.00	0.00	0.00	0.00	1.07	0.04
0.4	0.5	1.06	0.02	0.00	0.00	0.00	0.00	1.06	0.02
0.4	0.6	1.08	0.06	0.00	0.00	0.00	0.00	1.08	0.06
0.4	0.8	1.18	0.06	0.00	0.00	0.00	0.00	1.18	0.06
0.4	1	1.13	0.10	0.00	0.00	0.00	0.00	1.13	0.10
0.5	0	0.20	0.01	0.23	0.01	0.00	0.00	0.43	0.03
0.5	0.1	1.10	0.03	0.00	0.00	0.00	0.00	1.10	0.03
0.5	0.2	1.17	0.07	0.00	0.00	0.00	0.00	1.17	0.07
0.5	0.3	1.35	0.13	0.00	0.00	0.00	0.00	1.35	0.13
0.5	0.4	1.30	0.07	0.00	0.00	0.00	0.00	1.30	0.07
0.5	0.5	1.30	0.10	0.00	0.00	0.00	0.00	1.30	0.10
0.5	0.6	1.32	0.04	0.00	0.00	0.00	0.00	1.32	0.04
0.5	0.8	1.39	0.10	0.00	0.00	0.00	0.00	1.39	0.10
0.5	1	1.27	0.22	0.00	0.00	0.00	0.00	1.27	0.22
0.6	0	0.26	0.02	0.29	0.02	0.00	0.00	0.56	0.05
0.6	0.1	1.35	0.09	0.00	0.00	0.00	0.00	1.35	0.09
0.6	0.2	1.45	0.05	0.00	0.00	0.00	0.00	1.45	0.05
0.6	0.3	1.46	0.16	0.00	0.00	0.00	0.00	1.46	0.16
0.6	0.4	1.52	0.11	0.00	0.00	0.00	0.00	1.52	0.11
0.6	0.5	1.52	0.06	0.00	0.00	0.00	0.00	1.52	0.06

0.6	0.6	1.75	0.18	0.00	0.00	0.00	0.00	1.75	0.18
0.6	0.8	1.66	0.20	0.00	0.00	0.00	0.00	1.66	0.20
0.6	1	1.49	0.04	0.00	0.00	0.00	0.00	1.49	0.04
0.7	0	0.29	0.04	0.27	0.03	0.00	0.00	0.57	0.07
0.7	0.1	1.50	0.13	0.00	0.00	0.00	0.00	1.50	0.13
0.7	0.2	1.51	0.15	0.00	0.00	0.00	0.00	1.51	0.15
0.7	0.3	1.53	0.09	0.00	0.00	0.00	0.00	1.53	0.09
0.7	0.4	1.58	0.05	0.00	0.00	0.00	0.00	1.58	0.05
0.7	0.5	1.46	0.08	0.00	0.00	0.00	0.00	1.46	0.08
0.7	0.6	0.87	0.61	0.00	0.00	0.00	0.00	0.87	0.61
0.7	0.8	1.14	0.48	0.00	0.00	0.00	0.00	1.14	0.48
0.7	1	1.72	0.38	0.00	0.00	0.00	0.00	1.72	0.38
0.8	0	0.34	0.02	0.33	0.01	0.00	0.00	0.67	0.03
0.8	0.1	1.52	0.05	0.00	0.00	0.00	0.00	1.52	0.05
0.8	0.2	1.60	0.39	0.00	0.00	0.00	0.00	1.61	0.40
0.8	0.3	1.80	0.02	0.00	0.00	0.00	0.00	1.80	0.02
0.8	0.4	1.80	0.08	0.00	0.00	0.00	0.00	1.80	0.08
0.8	0.5	1.56	0.24	0.00	0.00	0.00	0.00	1.56	0.24
0.8	0.6	1.78	0.09	0.00	0.00	0.00	0.00	1.79	0.09
0.8	0.8	1.68	0.01	0.00	0.00	0.00	0.00	1.68	0.01
0.8	1	1.82	0.13	0.00	0.00	0.00	0.00	1.82	0.13
0.9	0	0.36	0.01	0.33	0.01	0.00	0.00	0.68	0.01
0.9	0.1	1.58	0.30	0.00	0.00	0.00	0.00	1.58	0.30
0.9	0.2	1.80	0.14	0.00	0.00	0.00	0.00	1.80	0.14
0.9	0.3	2.03	0.09	0.00	0.00	0.00	0.00	2.03	0.09
0.9	0.4	1.95	0.09	0.00	0.00	0.00	0.00	1.95	0.10
0.9	0.5	1.85	0.13	0.00	0.00	0.00	0.00	1.85	0.13
0.9	0.6	1.91	0.05	0.00	0.00	0.00	0.00	1.91	0.05
0.9	0.8	1.56	0.85	0.00	0.00	0.00	0.00	1.56	0.85
0.9	1	1.60	0.23	0.00	0.00	0.00	0.00	1.60	0.23
1	0	0.46	0.03	0.38	0.02	0.00	0.00	0.84	0.05
1	0.1	1.81	0.07	0.00	0.00	0.00	0.00	1.81	0.07
1	0.2	1.83	0.25	0.00	0.00	0.00	0.00	1.83	0.25
1	0.3	1.86	0.30	0.00	0.00	0.00	0.00	1.86	0.30
1	0.4	1.96	0.07	0.00	0.00	0.00	0.00	1.96	0.07
1	0.5	1.94	0.07	0.00	0.00	0.00	0.00	1.94	0.07
1	0.6	2.13	0.07	0.00	0.00	0.00	0.00	2.13	0.07
1	0.8	2.11	0.08	0.00	0.00	0.00	0.00	2.11	0.08
1	1	2.23	0.24	0.00	0.00	0.00	0.00	2.23	0.24
1.2	0	0.55	0.02	0.40	0.02	0.00	0.00	0.95	0.04
1.2	0.1	2.16	0.08	0.00	0.00	0.00	0.00	2.16	0.08
1.2	0.2	2.11	0.05	0.00	0.00	0.00	0.00	2.11	0.05
1.2	0.3	2.28	0.10	0.00	0.00	0.00	0.00	2.29	0.10
1.2	0.4	2.25	0.05	0.00	0.00	0.00	0.00	2.25	0.05
1.2	0.5	2.30	0.05	0.00	0.00	0.00	0.00	2.31	0.05
1.2	0.6	2.32	0.13	0.00	0.00	0.00	0.00	2.32	0.13
1.2	0.8	2.18	0.09	0.00	0.00	0.00	0.00	2.18	0.10
1.2	1	2.26	0.11	0.00	0.00	0.00	0.00	2.26	0.11
1.4	0	0.59	0.03	0.39	0.01	0.00	0.00	0.98	0.04
1.4	0.1	2.21	0.15	0.00	0.00	0.00	0.00	2.21	0.15
1.4	0.2	2.37	0.29	0.00	0.00	0.00	0.00	2.37	0.29
1.4	0.3	2.22	0.11	0.00	0.00	0.00	0.00	2.22	0.11
1.4	0.4	2.25	0.22	0.00	0.00	0.00	0.00	2.26	0.22
1.4	0.5	2.57	0.10	0.00	0.00	0.00	0.00	2.57	0.10
1.4	0.6	2.49	0.01	0.00	0.00	0.00	0.00	2.49	0.01
1.4	0.8	2.49	0.04	0.00	0.00	0.00	0.00	2.49	0.04
1.4	1	2.43	0.06	0.00	0.00	0.00	0.00	2.44	0.06
1.6	0	0.74	0.03	0.41	0.01	0.00	0.00	1.15	0.04
1.6	0.1	2.41	0.16	0.00	0.00	0.00	0.00	2.41	0.16
1.6	0.2	2.46	0.04	0.00	0.00	0.00	0.00	2.46	0.04
1.6	0.3	2.49	0.15	0.00	0.00	0.00	0.00	2.49	0.15
1.6	0.4	2.41	0.10	0.00	0.00	0.00	0.00	2.41	0.10
1.6	0.5	2.70	0.30	0.00	0.00	0.00	0.00	2.71	0.31
1.6	0.6	2.63	0.09	0.00	0.00	0.00	0.00	2.63	0.09
1.6	0.8	2.63	0.16	0.00	0.00	0.00	0.00	2.63	0.16

1.6	1	2.58	0.03	0.00	0.00	0.00	0.00	2.58	0.03
1.8	0	0.81	0.05	0.43	0.03	0.00	0.00	1.24	0.08
1.8	0.1	2.56	0.07	0.00	0.00	0.00	0.00	2.56	0.07
1.8	0.2	2.55	0.07	0.00	0.00	0.00	0.00	2.55	0.07
1.8	0.3	2.57	0.21	0.00	0.00	0.00	0.00	2.57	0.21
1.8	0.4	2.71	0.17	0.00	0.00	0.00	0.00	2.71	0.17
1.8	0.5	2.66	0.02	0.00	0.00	0.00	0.00	2.66	0.02
1.8	0.6	2.60	0.05	0.00	0.00	0.00	0.00	2.60	0.05
1.8	0.8	2.54	0.07	0.00	0.00	0.00	0.00	2.54	0.07
1.8	1	2.65	0.20	0.00	0.00	0.00	0.00	2.65	0.20
2	0	0.87	0.04	0.45	0.02	0.00	0.00	1.32	0.06
2	0.1	2.51	0.11	0.00	0.00	0.00	0.00	2.52	0.11
2	0.2	2.57	0.23	0.00	0.00	0.00	0.00	2.57	0.23
2	0.3	2.66	0.08	0.00	0.00	0.00	0.00	2.66	0.08
2	0.4	2.62	0.26	0.00	0.00	0.00	0.00	2.62	0.26
2	0.5	2.73	0.12	0.00	0.00	0.00	0.00	2.73	0.12
2	0.6	2.66	0.09	0.00	0.00	0.00	0.00	2.66	0.09
2	0.8	2.01	0.37	0.00	0.00	0.00	0.00	2.01	0.37
2	1	2.87	0.13	0.00	0.00	0.00	0.00	2.87	0.13

Reported values represent average and standard deviation for PET constituent monomers released during reactions performed in triplicate over 96 h at 30°C.

Table S5. Putative protocatechuate-dioxygenases in *Hydrogenophaga* sp. PML113 and *Comamonas thiooxydans*

Organism	Query	Hit Accession Numbers	% Identity	E-value	Bit score
Hydrogenophaga sp. PML113	LigA	WP_070398564.1	67.2	2.34E-49	166
		WP_070400956.1	43.1	8.31E-27	101
	LigB	WP_070398565.1	63.0	1.35E-122	384
		WP_070400957.1	56.6	5.73E-114	359
Comamonas thiooxydans DS1	LigA	KGH27325.1	65.0	1.25E-46	158
		KGH19511.1	61.7	4.68E-44	151
	LigB	KGH23198.1	62.1	1.45E-76	252
Comamonas thiooxydans DF1	LigA	KGH27550.1	65.0	1.23E-46	158
		KGH13041.1	61.7	4.6E-44	151
	LigB	KGH19836.1 (partial sequence)	69.9	3.14E-56	175
		KGH19529.1 (partial sequence)	62.8	2.23E-47	152
Comamonas thiooxydans DF2	LigA	KGH19350.1	65.0	1.24E-46	158
		KGH20562.1	61.7	4.66E-44	151
	LigB	KGH20561.1	62.3	5.18E-119	374
		KGH19470.1 (partial sequence)	61.4	8E-60	204

Putative protocatechuate (PCA)-dioxygenases in *Hydrogenophaga* sp. PML113 and *Comamonas thiooxydans* strains DS1, DF1, and DF2. PCA-2,3-dioxygenase from *Paenibacillus* sp. JJ-1b (PraA, accession number BAH79099.1), PCA-3,4-dioxygenase alpha and beta subunits from *Pseudomonas putida* KT2440 (PcaH and PcaG, accession numbers WP_010955312.1 and WP_009682255.1) and PCA-4,5-dioxygenase alpha and beta subunits from *Sphingobium* sp. SYK-6 (LigA and LigB, accession numbers BAK65924.1 and BAK65925.1) were used as query. Only hits with >100 bit score from tblastn searches against whole-genome sequences are shown.

Table S6. Summary of conditions tested for quenching MHETase enzymatic activity

Quenching solution	Non-enzymatic hydrolysis of MHET (%)		Enzyme activity quenched?	
	**No heat treatment**	**85°C, 10 min**	**No heat treatment**	**85°C, 10 min**
20% (v/v) DMSO mixed with 80% (v/v) Buffer Q: 100 mM NaCl, 200 mM sodium phosphate, pH 2.5	0	0	No	No
20% (v/v) DMSO, 80 mM NaCl, 160 mM sodium phosphate, pH 2.5	0	0	No	No
6N HCl, 50% DMSO	4.6	39.4	Unknown (high levels of acidolysis)	Unknown (high levels of acidolysis)
100% methanol	0.18	0.25	Yes	Yes
95% ethanol	0	0.69	Yes (causes precipitation)	Yes (causes precipitation)
100% DMSO	0	0	No	No
100 nM PMSF in 100% DMSO	0	1.3	No	Inconsistent
10 mM TCEP in H_2O	0	0.14	No	No
6M GuHCl	0	0.62	No	No
100 nM PMSF in 100% isopropanol	0	0.54	Inconsistent	Inconsistent
6M GuHCl, 10 mM TCEP	0	0.37	No	No

Summary of trial experiments performed in triplicate to determine the most satisfactory method for quenching MHETase enzymatic activity. Experiments were performed in reaction buffer (250 µM MHET, 90 mM NaCl, 10 % (v/v) DMSO, 45 mM sodium phosphate, pH 7.5) and quenched by addition of equal volume of the described quenching solution. The selected quenching method, using 100% methanol and 10 min heat treatment at 85°C, is indicated in grey.

Supplementary Movies

Movie S1. MHETase acylation reaction. Aimless Shooting trajectory demonstrating the basic elements of the acylation reaction of MHET by *I. sakaiensis* MHETase. In acylation, the acyl-enzyme intermediate (AEI) is formed, and ethylene glycol is released. MHET substrate is shown in purple-colored sticks, and the catalytic residues (His528, Asp492, and nucleophile Ser225) are shown in white sticks. MHETase backbone is shown in transparent white cartoon. Total simulation time shown is 2 ps. These path sampling simulations constitute completely unrestrained instances of the chemical reaction and were undertaken purely for illustrative purposes. Most hydrogens not directly involved in the reaction are not shown for clarity; the one exception is the hydrogen on His528 that hydrogen bonds with Asp492 throughout acylation. Movie was created in VMD (57).

Movie S2. Release of acylation product ethylene glycol. Molecular dynamics (MD) trajectory of the acyl-enzyme intermediate (AEI) immediately following the acylation reaction. The AEI features a covalent bond between the nucleophile Ser225 and the terephthalic acid moiety of MHET. Upon leaving the enzyme active site, EG is fully immersed in water, which is present in the simulation but not shown for clarity. Color schemes and representations are the same as in Supplementary Movie S1, with the exception that the protein backbone shown in cartoon is now opaque. The simulation length is 1 ns and is performed with a purely classical forcefield (no QM region). 500 frames from this simulation are utilized in the movie; coordinates for the cartoon protein backbone are smoothed over 6 frames, while coordinates for the stick representations are smoothed over 4 frames. Movie was created in VMD (57).

Movie S3. MHETase deacylation reaction. Aimless Shooting trajectory demonstrating the basic elements of the deacylation reaction. The acyl-enzyme intermediate (AEI) formed in the acylation reaction is broken and terephthalic acid (TPA) is released. The catalytic residues Ser225 and His528 are regenerated in this step and TPA released. Color schemes and representations are the same as in Supplementary Movie S1. Total simulation time shown is 2 ps. Most hydrogens not directly involved in the reaction are not shown for clarity; the one exception is the hydrogen on His528 that hydrogen bonds with Asp492 throughout deacylation. Movie was created in VMD (57).

Supplementary Data (Excel file)

Table SD1. Plasmid construction. Details are provided for the 21 plasmid constructs used in this study, including protein description, plasmid description and construction details, references and additional notes.

Table SD2. Synthesized DNA fragments. The full sequence is provided for the synthetic DNA fragments generated for this study with descriptions.

Table SD3. Primers. The full sequence is provided for all the oligonucleotide DNA primers generated for this study with descriptions.

Supplementary References

1. F. W. Studier, Protein production by auto-induction in high density shaking cultures. *Protein Expr. Purif.* **41**, 207-234 (2005).
2. P. K. Smith *et al.*, Measurement of protein using bicinchoninic acid. *Anal. Biochem.* **150**, 76-85 (1985).
3. F. C. Neidhardt, P. L. Bloch, D. F. Smith, Culture medium for enterobacteria. *J. Bacteriol.* **119**, 736-747 (1974).
4. W. Kabsch, XDS. *Acta Cryst. D* **66**, 125-132 (2010).
5. Anonymous, The CCP4 suite: programs for protein crystallography. *Acta Cryst. D* **50**, 760-763 (1994).
6. A. J. McCoy *et al.*, Phaser crystallographic software. *J. Appl. Crystallogr.* **40**, 658-674 (2007).
7. G. M. Sheldrick, Experimental phasing with SHELXC/D/E: combining chain tracing with density modification. *Acta Cryst. D* **66**, 479-485 (2010).
8. P. D. Adams *et al.*, PHENIX: a comprehensive Python-based system for macromolecular structure solution. *Acta Cryst. D* **66**, 213-221 (2010).
9. P. V. Afonine *et al.*, Towards automated crystallographic structure refinement with phenix.refine. *Acta Cryst. D* **68**, 352-367 (2012).
10. P. Emsley, K. Cowtan, Coot: model-building tools for molecular graphics. *Acta Cryst. D* **60**, 2126-2132 (2004).
11. R. A. Engh, R. Huber, Accurate bond and angle parameters for X-ray protein structure refinement. *Acta Cryst. A* **47**, 392-400 (1991).
12. M. A. Larkin *et al.*, Clustal W and Clustal X version 2.0. *Bioinformatics (Oxford, England)* **23**, 2947-2948 (2007).
13. X. Robert, P. Gouet, Deciphering key features in protein structures with the new ENDscript server. *Nucleic Acid Res.* **42**, W320-W324 (2014).
14. G. J. Palm *et al.*, Structure of the plastic-degrading *Ideonella sakaiensis* MHETase bound to a substrate. *Nature Comm.* **10**, 1717 (2019).
15. S. Yoshida *et al.*, A bacterium that degrades and assimilates poly(ethylene terephthalate). *Science (New York, N.Y.)* **351**, 1196-1199 (2016).
16. K. Katoh, D. M. Standley, MAFFT multiple sequence alignment software version 7: improvements in performance and usability. *Mol. Biol. Evol.* **30**, 772-780 (2013).
17. J. A. Capra, M. Singh, Predicting functionally important residues from sequence conservation. *Bioinformatics (Oxford, England)* **23**, 1875-1882 (2007).
18. P. J. Cock *et al.*, Biopython: freely available Python tools for computational molecular biology and bioinformatics. *Bioinformatics (Oxford, England)* **25**, 1422-1423 (2009).
19. S. R. Eddy, Profile hidden Markov models. *Bioinformatics (Oxford, England)* **14**, 755-763 (1998).
20. S. Kumar, G. Stecher, K. Tamura, MEGA7: Molecular Evolutionary Genetics Analysis Version 7.0 for Bigger Datasets. *Mol. Biol. Evol.* **33**, 1870-1874 (2016).
21. D. T. Jones, W. R. Taylor, J. M. Thornton, The rapid generation of mutation data matrices from protein sequences. *Comput. Appl. Biosci.* **8**, 275-282 (1992).
22. M. Nei, S. Kumar, *Molecular evolution and phylogenetics* (Oxford university press, 2000).
23. N. Saitou, M. Nei, The neighbor-joining method: a new method for reconstructing phylogenetic trees. *Mol. Biol. Evol.* **4**, 406-425 (1987).
24. Anonymous, Schrödinger Release 2017-3: Schrödinger Suite 2017-3 Protein Preparation Wizard.
25. Anonymous, Schrödinger Release 2017-3: Impact, Schrödinger, LLC, New York, NY, 2016.
26. G. M. Sastry, M. Adzhigirey, T. Day, R. Annabhimoju, W. Sherman, Protein and ligand preparation: parameters, protocols, and influence on virtual screening enrichments. *J. Comput. Aided Mol. Des.* **27**, 221-234 (2013).
27. J. L. Banks *et al.*, Integrated Modeling Program, Applied Chemical Theory (IMPACT). *J. Comput. Chem.* **26**, 1752-1780 (2005).
28. Anonymous, Schrödinger Release 2019-1: LigPrep, Schrödinger, LLC, New York, NY, 2019.
29. J. R. Greenwood, D. Calkins, A. P. Sullivan, J. C. Shelley, Towards the comprehensive, rapid, and accurate prediction of the favorable tautomeric states of drug-like molecules in aqueous solution. *J. Comput. Aided Mol. Des.* **24**, 591-604 (2010).
30. Anonymous, Schrödinger Suite 2018-4 Induced Fit Docking protocol; Glide, Schrödinger, LLC, New York, NY, 2018; Prime, Schrödinger, LLC, New York, NY, 2018.
31. J. C. Shelley *et al.*, Epik: a software program for pK(a) prediction and protonation state generation for drug-like molecules. *J. Comput. Aided Mol. Des.* **21**, 681-691 (2007).
32. R. Farid, T. Day, R. A. Friesner, R. A. Pearlstein, New insights about HERG blockade obtained from protein modeling, potential energy mapping, and docking studies. *Bioorg. Med. Chem.* **14**, 3160-3173 (2006).
33. W. Sherman, H. S. Beard, R. Farid, Use of an induced fit receptor structure in virtual screening. *Chem. Biol. Drug Des.* **67**, 83-84 (2006).
34. W. Sherman, T. Day, M. P. Jacobson, R. A. Friesner, R. Farid, Novel procedure for modeling ligand/receptor induced fit effects. *J. Med. Chem.* **49**, 534-553 (2006).
35. R. Anandakrishnan, B. Aguilar, A. V. Onufriev, H++ 3.0: automating p K prediction and the preparation of biomolecular structures for atomistic molecular modeling and simulations. *Nucleic Acid Res.* **40**, W537-W541 (2012).
36. B. R. Brooks *et al.*, CHARMM: the biomolecular simulation program. *J. Comp. Chem.* **30**, 1545-1614 (2009).
37. R. B. Best *et al.*, Optimization of the additive CHARMM all-atom protein force field targeting improved sampling of the backbone ϕ, ψ and side-chain χ1 and χ2 dihedral angles. *J. Chem. Theory Comput.* **8**, 3257-3273 (2012).
38. O. Guvench, E. Hatcher, R. M. Venable, R. W. Pastor, A. D. MacKerell, CHARMM Additive All-Atom Force Field for Glycosidic Linkages between Hexopyranoses. *J. Chem. Theory Comput.* **5**, 2353-2370 (2009).
39. O. Guvench *et al.*, CHARMM Additive All-Atom Force Field for Carbohydrate Derivatives and Its Utility in Polysaccharide and Carbohydrate–Protein Modeling. *J. Chem. Theory Comput.* **7**, 3162-3180 (2011).
40. W. L. Jorgensen, J. Chandrasekhar, J. D. Madura, R. W. Impey, M. L. Klein, Comparison of simple potential functions for simulating liquid water. *J. Chem. Phys.* **79**, 926-935 (1983).
41. K. Vanommeslaeghe, A. D. MacKerell, Automation of the CHARMM General Force Field (CGenFF) I: Bond Perception and Atom Typing. *J. Chem. Inf. Model* **52**, 3144-3154 (2012).
42. K. Vanommeslaeghe, E. P. Raman, A. D. MacKerell, Automation of the CHARMM General Force Field (CGenFF) II: Assignment of Bonded Parameters and Partial Atomic Charges. *J. Chem. Inf. Model* **52**, 3155-3168 (2012).
43. K. Vanommeslaeghe *et al.*, CHARMM general force field: A force field for drug-like molecules compatible with the CHARMM all-atom additive biological force fields. *J. Comp. Chem.* **31**, 671-690 (2010).
44. W. Yu, X. He, K. Vanommeslaeghe, A. D. MacKerell Jr, Extension of the CHARMM general force field to sulfonyl-containing compounds and its utility in biomolecular simulations. *J. Comp. Chem.* **33**, 2451-2468 (2012).
45. H. P. Austin *et al.*, Characterization and engineering of a plastic-degrading aromatic polyesterase. *Proc. Natl. Acad. Sci.* **115**, E4350-E4357 (2018).
46. J. C. Phillips *et al.*, Scalable molecular dynamics with NAMD. *J. Comp. Chem.* **26**, 1781-1802 (2005).

47. J.-P. Ryckaert, G. Ciccotti, H. J. C. Berendsen, Numerical integration of the cartesian equations of motion of a system with constraints: molecular dynamics of n-alkanes. *J. Comp. Phys.* **23**, 327-341 (1977).
48. M. F. Crowley, M. J. Williamson, R. C. Walker, CHAMBER: Comprehensive support for CHARMM force fields within the AMBER software. *Intl. J. Quantum Chem.* **109**, 3767-3772 (2009).
49. D. A. Case *et al.*, AMBER 12; University of California, San Francisco. (2012).
50. G. d. M. Seabra, R. C. Walker, M. Elstner, D. A. Case, A. E. Roitberg, Implementation of the SCC-DFTB Method for Hybrid QM/MM Simulations within the Amber Molecular Dynamics Package. *J. Phys. Chem. A* **111**, 5655-5664 (2007).
51. R. C. Walker, M. F. Crowley, D. A. Case, The implementation of a fast and accurate QM/MM potential method in Amber. *J. Comp. Chem.* **29**, 1019-1031 (2008).
52. M. Gaus, A. Goez, M. Elstner, Parametrization and benchmark of DFTB3 for organic molecules. *J. Chem. Theory Comput.* **9**, 338-354 (2013).
53. T.-S. Lee, B. K. Radak, A. Pabis, D. M. York, A new maximum likelihood approach for free energy profile construction from molecular simulations. *J. Chem. Theory Comput.* **9**, 153-164 (2013).
54. P. G. Bolhuis, D. Chandler, C. Dellago, P. L. Geissler, Transition path sampling: Throwing ropes over rough mountain passes, in the dark. *Annu. Rev. Phys. Chem.* **53**, 291-318 (2002).
55. B. Peters, G. T. Beckham, B. L. Trout, Extensions to the likelihood maximization approach for finding reaction coordinates. *J. Chem. Phys.* **127**, 034109 (2007).
56. B. Peters, B. L. Trout, Obtaining reaction coordinates by likelihood maximization. *J. Chem. Phys.* **125**, 054108 (2006).
57. W. Humphrey, A. Dalke, K. Schulten, VMD: visual molecular dynamics. *J. Mol. Graphics* **14**, 33-38 (1996).
58. S. Kumar, G. Stecher, M. Li, C. Knyaz, K. Tamura, MEGA X: Molecular Evolutionary Genetics Analysis across Computing Platforms. *Mol. Biol. Evol.* **35**, 1547-1549 (2018).
59. F. Sievers *et al.*, Fast, scalable generation of high-quality protein multiple sequence alignments using Clustal Omega. *Mol. Syst. Biol.* **7**, 539 (2011).
60. V. B. Chen *et al.*, MolProbity: all-atom structure validation for macromolecular crystallography. *Acta Cryst. D* **66**, 12-21 (2010).
